# Evolutionary Trajectory of the Replication Mode of Bacterial Replicons

**DOI:** 10.1128/mBio.02745-20

**Published:** 2021-01-26

**Authors:** Bin-Bin Xie, Jin-Cheng Rong, Bai-Lu Tang, Sishuo Wang, Guiming Liu, Qi-Long Qin, Xi-Ying Zhang, Weipeng Zhang, Qunxin She, Yin Chen, Fuchuan Li, Shengying Li, Xiu-Lan Chen, Haiwei Luo, Yu-Zhong Zhang

**Affiliations:** a State Key Laboratory of Microbial Technology, Marine Biotechnology Research Center, Shandong University, Qingdao, China; b College of Marine Life Sciences and Frontiers Science Center for Deep Ocean Multispheres and Earth System, Ocean University of China, Qingdao, China; c Laboratory for Marine Biology and Biotechnology, Pilot National Laboratory for Marine Science and Technology (Qingdao), Qingdao, China; d Simon F. S. Li Marine Science Laboratory, School of Life Sciences, and State Key Laboratory of Agrobiotechnology, The Chinese University of Hong Kong, Shatin, Hong Kong SAR; e Beijing Agro-Biotechnology Research Center, Beijing Academy of Agriculture and Forestry Sciences, Beijing, China; f School of Life Sciences, University of Warwick, Coventry, United Kingdom; University of Pittsburgh

**Keywords:** chromid, chromosome replication, unidirectional replication, chromosome evolution, *Pseudoalteromonas*

## Abstract

As typical bacterial replicons, circular chromosomes replicate bidirectionally and circular plasmids replicate either bidirectionally or unidirectionally. Whereas the finding of chromids (plasmid-derived chromosomes) in multiple bacterial lineages provides circumstantial evidence that chromosomes likely evolved from plasmids, all experimentally assayed chromids were shown to use bidirectional replication. Here, we employed a model system, the marine bacterial genus *Pseudoalteromonas*, members of which consistently carry a chromosome and a chromid. We provide experimental and bioinformatic evidence that while chromids in a few strains replicate bidirectionally, most replicate unidirectionally. This is the first experimental demonstration of the unidirectional replication mode in bacterial chromids. Phylogenomic and comparative genomic analyses showed that the bidirectional replication evolved only once from a unidirectional ancestor and that this transition was associated with insertions of exogenous DNA and relocation of the replication terminus region (*ter2*) from near the origin site (*ori2*) to a position roughly opposite it. This process enables a plasmid-derived chromosome to increase its size and expand the bacterium’s metabolic versatility while keeping its replication synchronized with that of the main chromosome. A major implication of our study is that the uni- and bidirectionally replicating chromids may represent two stages on the evolutionary trajectory from unidirectionally replicating plasmids to bidirectionally replicating chromosomes in bacteria. Further bioinformatic analyses predicted unidirectionally replicating chromids in several unrelated bacterial phyla, suggesting that evolution from unidirectionally to bidirectionally replicating replicons occurred multiple times in bacteria.

## Abstract (precis)

Chromosome replication is an essential process for cell division. The mode of chromosome replication has important impacts on the structure of the chromosome and replication speed.

## INTRODUCTION

Prokaryotic DNA replication has been well studied in model bacteria, including the Gram-negative organism Escherichia coli and the Gram-positive organism Bacillus subtilis, both of which have a single circular chromosome that replicates bidirectionally (1–3). In general, this bidirectional replication is initiated at the origin (*ori*) site, after which two replication forks proceed in opposite directions and ultimately terminate in the terminus (*ter*) region, located roughly opposite the *ori* site on the circular chromosome (see Fig. S1a and b in the supplemental material). DnaA, the bacterial replication initiator protein, commences replication by binding to the *ori* site; the Tus protein terminates replication by binding to *ter* sites in E. coli (4). Therefore, with the *ori* and *ter* regions, the chromosome is divided into two halves, called replichores (5).

10.1128/mBio.02745-20.1 FIG S1 The chromosome structure for model bacteria Escherichia coli (a), Bacillus subtilis (b), and Vibrio cholerae (c). Download FIG S1, PDF file, 0.5 MB. Copyright © 2021 Xie et al. 2021 Xie et al. This content is distributed under the terms of the Creative Commons Attribution 4.0 International license.

About 10% of bacteria contain more than one chromosome (6, 7). As exemplified in Vibrio cholerae, the causative agent of cholera, the secondary chromosomes have a nucleotide composition close to that of the main chromosome and frequently carry core genes that are found on the (main) chromosome in other species (8). They often use plasmid-type maintenance and replication systems and are therefore referred to as the chromids (9). Similar to the main chromosomes, the chromids of Vibrio cholerae replicate bidirectionally: the replication is initiated from the origin of the chromid (*ori2*), and two replication forks proceed in the opposite directions (10) (Fig. S1c).

Chromosome replication exerts genome-wide mutational and selective pressures (11–15). The leading and lagging strands of the replication fork replicate differently, resulting in different mutational patterns (16, 17). One such strand-dependent compositional asymmetry is called GC skew, which means higher frequency of guanines (Gs) than cytosines (Cs) on the leading strand (16, 18). For bidirectional replication of typical bacterial circular chromosomes, GC skew shows a bipartite pattern with two reflection points, one located near *ori* and the other near *ter*. A study revealed that the secondary chromosome (Chr2) of the marine bacterium Pseudoalteromonas haloplanktis TAC125 does not show an otherwise expected GC skew, suggesting that it may be replicated unidirectionally (19).

Members of the genus *Pseudoalteromonas* are ubiquitous in a variety of marine habitats (20, 21). These bacteria utilize a wide spectrum of nutrients, including insoluble polysaccharides (22–24), proteins (25, 26), and bacterial cell wall (27). They are also known to produce a large variety of biologically active natural products (e.g., antimicrobial, antifouling, and algicidal substances) (20, 28). Compared to the oligotrophic paradigm, such as members of the SAR11 bacteria, which consistently have streamlined genomes and grow under exceedingly low nutrient conditions (29), *Pseudoalteromonas* spp. are typical copiotrophs which harbor large and variable genomes, explore nutrient patches, and interact with eukaryotic hosts (23, 30). Here, the *Pseudoalteromonas* Chr2 was employed as a model system to investigate the versatility and evolution of bacterial chromosome replication by using a combination of genome sequencing, phylogenomic and comparative genomic analyses, and experimental assays. Our study reveals a definitive example of how an evolutionary transition from unidirectional to bidirectional replication allows the increase of the sizes of chromids, thereby illustrating a process through which plasmids evolve into chromosomes.

## RESULTS

### Survey of replication directions using complete publicly available bacterial genomes.

**FIG 1:**
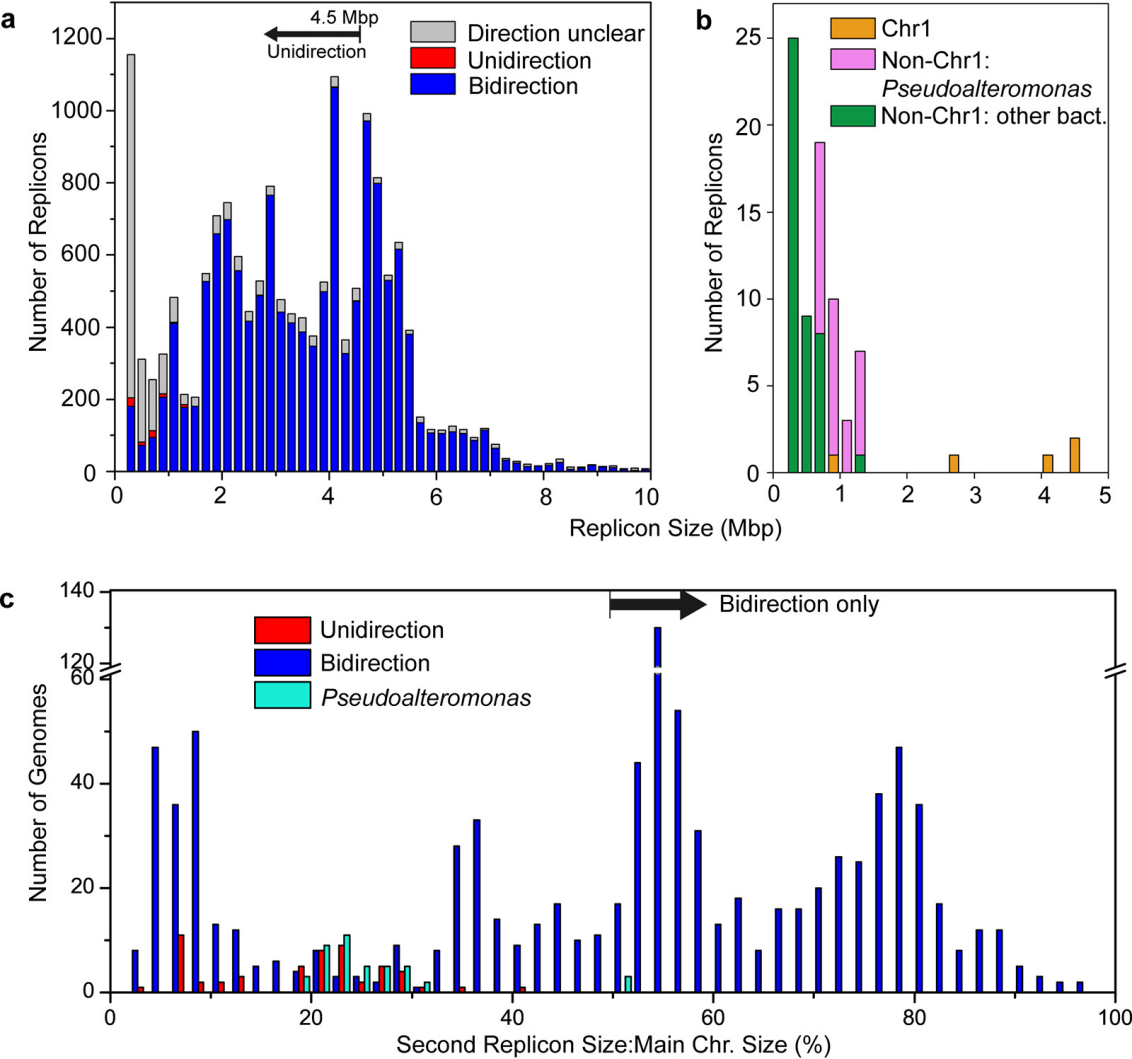
Survey of replication directions for publicly available complete bacterial genomes. (a) Prediction of replication direction of large replicons (>200 kb) based on GC skew analyses. (b) Predicted unidirectionally replicating replicons. (c) Comparison of the ratio of Chr2 size to Chr1 size for bidirectionally and unidirectionally replicating Chr2 from bacteria with multiple chromosomes and Chr2 from *Pseudoalteromonas*.

To search for bacterial chromosomes that might replicate unidirectionally, GC skew was calculated for all complete bacterial genomes available from the NCBI RefSeq database. By using the GC skew patterns of E. coli, B. subtilis, and V. cholerae chromosomes as the standard for bidirectional replication and that of the P. haloplanktis TAC125 chromid as the standard for unidirectional replication, replication directions were predicted for 16,103 large replicons (>200 kb) out of the total 26,371 replicons from 13,550 genomes. Among them, 2,304 were unpredictable and 13,722 were predicted to replicate bidirectionally and 77 unidirectionally (Fig. 1a; also, see Table S1). Results showed that the unidirectionally replicating large replicons may exist in different phyla, including *Proteobacteria* (*Gammaproteobacteria* and *Alphaproteobacteria*), *Firmicutes*, and *Bacteroidetes* (Table S1). Among these, the genus *Pseudoalteromonas* (order *Alteromonadales* of the class *Gammaproteobacteria*) was the major group that likely harbored a unidirectionally replicating secondary replicon (Fig. 1b; Table S1).

10.1128/mBio.02745-20.7 TABLE S1 Predicted unidirectionally replicating replicons (>200 kb) from NCBI RefSeq database based on GC skew. Download Table S1, PDF file, 0.3 MB. Copyright © 2021 Xie et al. 2021 Xie et al. This content is distributed under the terms of the Creative Commons Attribution 4.0 International license.

### Genome sequencing of *Pseudoalteromonas* spp.

To investigate the unidirectional chromosome replication in *Pseudoalteromonas*, we collected type strains of the 26 species in this genus, which accounted for more than half of the currently recognized species of the genus. Using a combination of Illumina sequencing and PCR gap-closing, we obtained complete genome sequences for nine strains (Table S2). Similar to P. haloplanktis TAC125, all nine strains with complete genome sequences had two circular chromosomes, 1 and 2. For the 13 of the 17 strains with draft genome sequences, we obtained the complete sequences for the circular Chr2. The remaining four strains were also predicted to have a Chr2, because their genome assemblies contain contigs that showed good colinearity with the complete Chr2. Therefore, our study suggests that all *Pseudoalteromonas* spp. had two circular chromosomes.

10.1128/mBio.02745-20.8 TABLE S2 Detailed information for 26 *Pseudoalteromonas* type strains sequenced in this study. Download Table S2, PDF file, 0.3 MB. Copyright © 2021 Xie et al. 2021 Xie et al. This content is distributed under the terms of the Creative Commons Attribution 4.0 International license.

### The replication system of *Pseudoalteromonas*.

**FIG 2:**
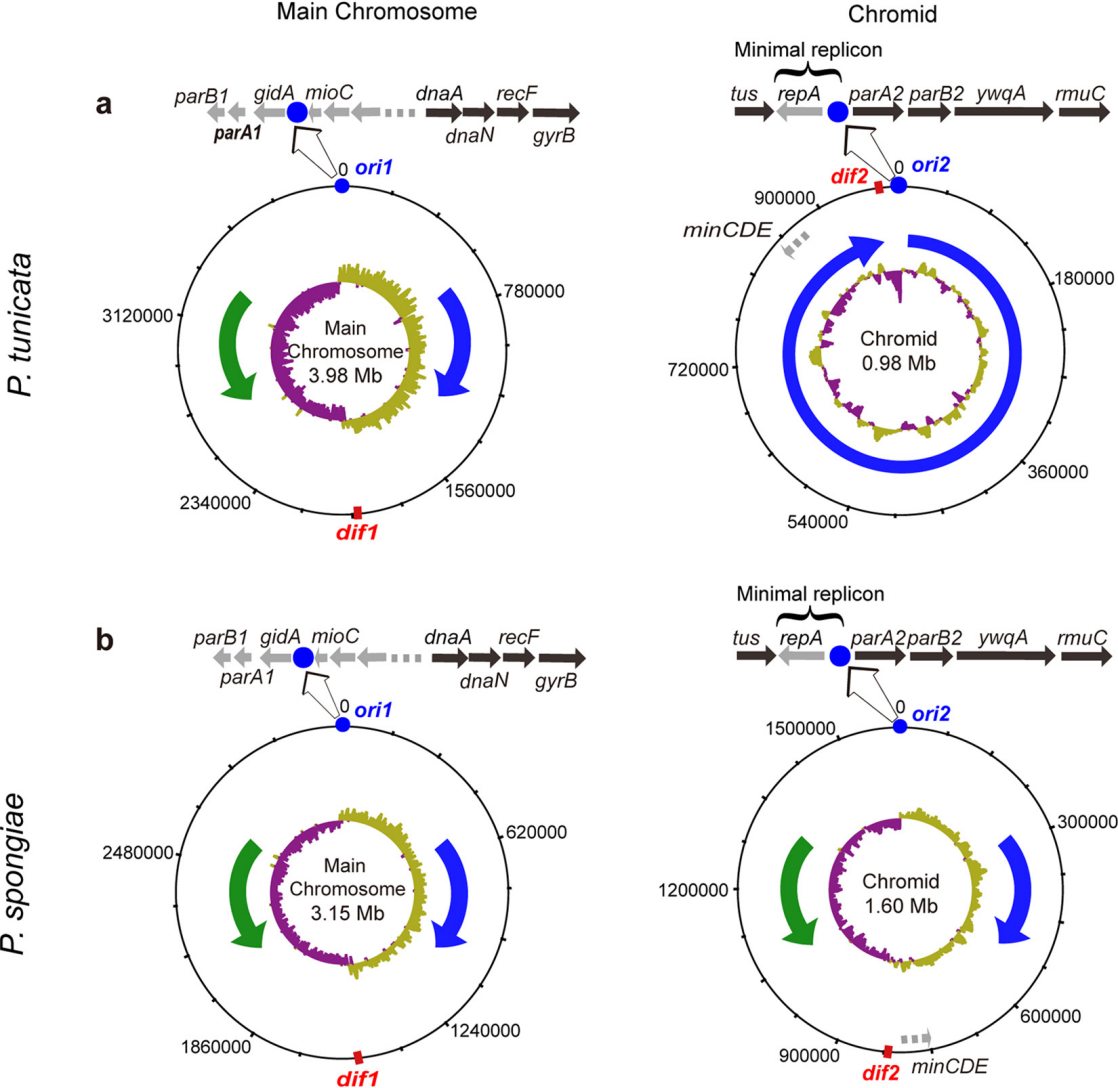
Comparison of the main chromosome and chromid structure for species *P. tunicata* (a) and *P. spongiae* (b). The *ori* site (blue circle), *dif* site (red rectangle), and genes related to main chromosome/chromid replication and maintenance are indicated on the outer circle. GC skew is shown as the inner circle. Predicted replication directions are shown with blue (clockwise) and green (counterclockwise) arrows.

Genomic analyses revealed a conserved replication system that contains genes encoding the replication terminus site-binding protein (*tus*), helicase (*dnaB*), DNA polymerase III subunits (*dnaE*, *dnaN*, *dnaQ*, *dnaX*, *holA*, *holB*, and *holC*), gyrase subunit B (*gyrB*), primase (*dnaG*), and the single-strand DNA-binding protein (*ssb*), all of which show high sequence similarities to those from E. coli (Fig. 2; Table S3a). The chromosome segregation system contains the structural maintenance of chromosomes (SMC) protein and the accessory segregation and condensation proteins ScpA and ScpB, all of which had homologs in B. subtilis but not in E. coli (Table S3a). In addition, genes *minCDE*, encoding septum site-determining proteins, were also annotated. These proteins are generally involved in plasmid segregation in E. coli and B. subtilis (for a review, see reference 4). Thus, it is likely that they are involved in the segregation of Chr2 in *Pseudoalteromonas*. While most of these genes were located on Chr1 in *Pseudoalteromonas*, the *tus* and *minCDE* genes, which were located on the circular chromosome in E. coli, were invariably on the Chr2 of *Pseudoalteromonas* (Fig. 2). Localization of the *tus* gene on the chromid of *Pseudoalteromonas* was reported previously (31).

10.1128/mBio.02745-20.9 TABLE S3 (a) Genes involved in chromosome replication and their homologues in model organisms V. cholerae, E. coli, and B. subtilis. (b) Replication origin sites (*ori*) and termination sites (*dif*) on the main chromosomes and chromids. Download Table S3, PDF file, 0.5 MB. Copyright © 2021 Xie et al. 2021 Xie et al. This content is distributed under the terms of the Creative Commons Attribution 4.0 International license.

Consistent with the fact that *Pseudoalteromonas* spp. have two chromosomes, two chromosome partitioning systems were identified in their genomes: *parA1B1* on Chr1 and *parA2B2* on Chr2. The *parA2B2* genes were located adjacent to the putative *ori2* site in a conserved gene cluster, which also contained the *tus* gene, the putative replication initiator protein gene *repA*, the possible DNA helicase gene *ywqA*, and the DNA recombination protein gene *rmuC* (Fig. 2). However, the functions of the *repA* gene and the replication origin *ori2* have not been tested experimentally. Here, we conducted a series of truncation experiments to identify the minimal replicon of the *Pseudoalteromonas* chromid using Pseudoalteromonas spongiae JCM 12884^T^ and Pseudoalteromonas tunicata DSM14096^T^ as representatives of the genus. Our results showed that *repA* together with the putative *ori2* site can initiate and are essential to the replication of a replicon lacking the replication initiator protein, confirming the function of RepA as the replication initiator protein and the predicted *ori2* site as the origin of Chr2 (Fig. 2 and Fig. S3). A sequence search against the NCBI nonredundant (nr) protein database with RepA as the query revealed no hits with significant identities in non-*Pseudoalteromonas* bacteria or plasmids, although it was suggested that it may be related to the initiator protein of the R1 plasmid (19). RepA also showed no detectable sequence similarity to the replication initiator protein DnaA encoded by Chr1. This result indicates that the Chr2 was likely derived from an unknown plasmid and, therefore, that Chr2 is a chromid. In sum, *Pseudoalteromonas* Chr1 and Chr2 have evolved different strategies to initiate the replication (DnaA+*ori1* versus RepA+*ori2*). As in *Pseudoalteromonas*, in V. cholerae the initiation of chromid replication is also different from that of the main chromosome, which uses DnaA to initiate the main chromosome replication and RctB to initiate the chromid replication (32).

### Unidirectional replication prevails among *Pseudoalteromonas* chromids.

Similar to the chromid of the strain TAC125, most *Pseudoalteromonas* chromids showed a GC skew different from that of a typical chromosome (Fig. 2a, right, and Fig. S4a), suggesting that unidirectional replication was prevalent among members of the *Pseudoalteromonas* genus. Another piece of evidence for unidirectional replication was related to the location of the *dif* site, which participates in chromosome segregation and often locates near the *ter* region (31, 33, 34). For the main chromosome, which replicated bidirectionally, the *dif* site (*dif1*) was located at a position roughly opposite *ori* (*ori1*) (Fig. 2 and Fig. S1). However, for most *Pseudoalteromonas* chromids (Table S3b), *dif2* was located adjacent to the *ori2* site (only approximately 4 to 27 kb away).

**FIG 3:**
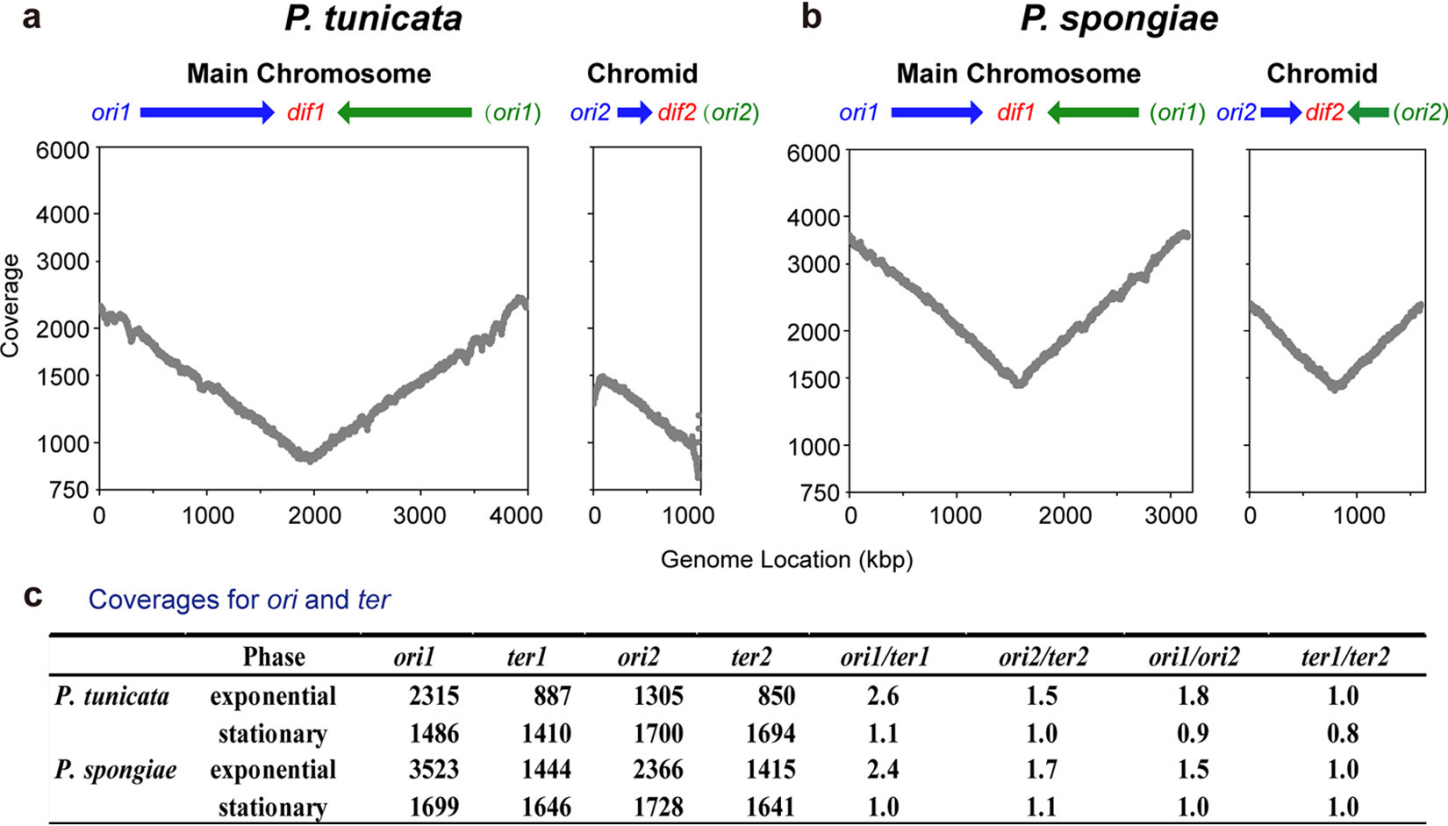
Analyses of replication direction and synchronization mode by deep genome sequencing. (a and b) Gradient changes of sequencing coverage from the replication origin (*ori* site) to the replication terminus (represented by *dif*) along a replicating replicon for *P. tunicata* DSM 14096^T^ (a) and *P. spongiae* JCM 12884^T^ (b) at the exponential phase. Data were presented in bins of 1,000 bp and were corrected using data from the stationary phase (see Materials and Methods). Note that the *y* axis was set to a base 2 logarithmic scale. Arrows indicate the replication direction (blue for clockwise and green for counterclockwise). (c) Sequencing coverage for the replication origin regions (*ori1* and *ori2*) and the terminus regions (*ter1* and *ter2*). Sequencing coverage of *ter1* and *ter2* was represented by the coverage near the *dif1* and *dif2* sites, respectively.

To experimentally determine the replication directions, we performed deep genome sequencing and analyzed the sequencing depth along the replicating main chromosomes and chromids. The underlying principle is that, once chromosome replication is initiated, the chromosomal region that has been replicated (i.e., near the *ori* site) should have an additional copy compared to the region that has not been replicated (i.e., near the *ter* site) (10, 35–37). Technically, this will be manifested as a higher coverage (i.e., read counts) for nucleotides near *ori* than those closer to *ter* (38). Our data showed that, at the exponential phase, the coverage decreased along Chr1 from *ori* to *dif* on both sides of *dif* (or on both replichores) (Fig. 3a and b), indicating that Chr1 was replicated bidirectionally. However, this symmetric pattern was missing from the *P. tunicata* chromid, indicating a unidirectional replication of this chromid (Fig. 3a). Similarly, deep sequencing also revealed unidirectional replication in seven other strains tested (Fig. S5a). This is the first experimental demonstration of the unidirectional replication of a bacterial chromosome.

### Presence of bidirectional replication in *Pseudoalteromonas* chromids.

Interestingly, among the *Pseudoalteromonas* species sequenced, *P. spongiae* harbored a chromid that showed a GC skew similar to that of the E. coli, B. subtilis, and V. cholerae chromosomes (Fig. 2b, right, and Fig. S1), suggesting that the *P. spongiae* chromid may replicate bidirectionally. Consistent with this hypothesis, the *dif2* site was located approximately opposite the *ori2* site on its chromid (Fig. 2b). Further support for bidirectional replication of the *P. spongiae* chromid was obtained from the deep-sequencing result, which showed a coverage profile highly similar to that of the bidirectionally replicating Chr1 (Fig. 3b). In addition, we collected experimental and/or bioinformatics evidence for bidirectional replication of the chromids in another two *Pseudoalteromonas* strains, including the strain SAO4-4 (39) (Fig. S4b) from our culture collection, as confirmed with the deep genome sequencing (Fig. S5b), and *P. piratica* OCN003^T^ (40) from the NCBI GenBank database (Fig. S4b). In sum, a total of only three strains among the currently available *Pseudoalteromonas* members carried bidirectionally replicating chromids, indicating that this replication mode appears to be less common than unidirectional replication among the *Pseudoalteromonas* chromids.

### Evolutionary history of the replication mode of *Pseudoalteromonas* chromids.

**FIG 4:**
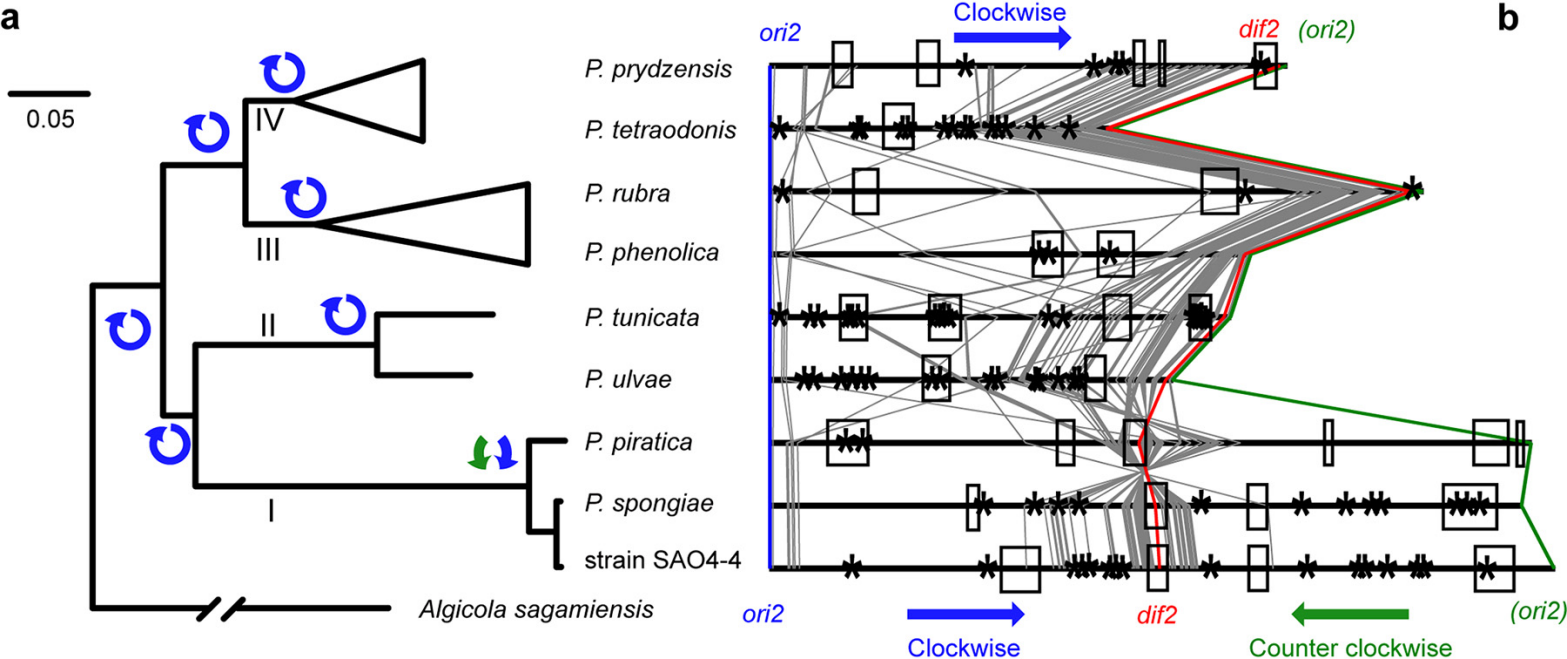
Phylogenomics analysis of *Pseudoalteromonas* spp. and comparison of the replicating directions of the chromids of different clades. (a) A maximum-likelihood tree reconstructed based on the concatenated amino acid sequences of shared single-copy genes on the main chromosome. The genus diverged into four major clades: I, II, III, and IV. The replication direction of the chromid was marked for all clades and their ancestors. All branches are supported by the IQ-Tree ultrafast bootstrap percentages of 100%. The bar represents 0.05 substitution per site. See details in Fig. S2a for the complete tree. (b) Colinear analysis of uni- and bidirectionally replicating chromids of *Pseudoalteromonas* spp. A total of 82 common single-gene families located on the chromids were found among the 43 genomes studied and are indicated using lines between chromids. Genes *parA2* and *parB2* and the locus *ori2* are shown in blue. Genes *repA* and *tus* are shown in green. The *ter2* region is represented by *dif2*. The locus *dif2* is shown in red. The remaining genes are shown in gray. It is clear that the neighboring region of *dif2* is highly conserved. Arrows indicate the replication directions. Asterisks indicate the predicted insertion sequences and integrons, and boxes indicate the predicted prophage-like regions.

To infer the evolutionary history of the replication direction of the chromids, we first attempted to construct a reliable species tree delineating the evolutionary history of the available *Pseudoalteromonas* members using phylogenomic approaches. Ideally, this species tree should be built with sequences from both the chromosome and the chromid of each strain. Unlike the main chromosome tree, where close relatives of *Pseudoalteromonas* were available to root the tree, however, there were no suitable outgroup sequences for the chromids. As a result, the phylogeny of the chromid was rooted using the less accurate strategy called midpoint rooting (Fig. S2b, d, and f). The identical topologies between the main chromosome tree (Fig. S2a, c, and e) and the chromid tree (Fig. S2b, d, and f) indicate that the main chromosome and the chromid shared the same evolutionary history since their origins. We did notice that one of the chromid trees (Topo 2) (Fig. S2d) differed from all other trees (Topo 1) (Fig. S2). We therefore statistically compared the two topologies (Topo 1 versus Topo 2) with sequences from the main chromosome and showed that Topo 1 was unanimously supported (Fig. S2g). Taken together, these lines of evidence support the idea that the main chromosome tree can represent the species tree (Fig. 4a).

10.1128/mBio.02745-20.2 FIG S2 Maximum-likelihood trees based on the concatenated amino acid sequences of single-copy genes on the main chromosome (a, c, and e) and chromid (b, d, and f) based on different alignment trimming strategies and the removal of compositionally heterogeneous genes. (g) Topology tests on two different topologies for the chromid trees. Download FIG S2, PDF file, 0.9 MB. Copyright © 2021 Xie et al. 2021 Xie et al. This content is distributed under the terms of the Creative Commons Attribution 4.0 International license.

10.1128/mBio.02745-20.3 FIG S3 Determination of the minimal replicon for the chromids. Download FIG S3, PDF file, 0.9 MB. Copyright © 2021 Xie et al. 2021 Xie et al. This content is distributed under the terms of the Creative Commons Attribution 4.0 International license.

10.1128/mBio.02745-20.4 FIG S4 Unidirectional (a) and bidirectional (b) replication of *Pseudoalteromonas* chromids revealed by GC skew analyses. Download FIG S4, PDF file, 1.1 MB. Copyright © 2021 Xie et al. 2021 Xie et al. This content is distributed under the terms of the Creative Commons Attribution 4.0 International license.

10.1128/mBio.02745-20.5 FIG S5 (a) Unidirectional replication revealed by deep sequencing at the exponential phase for seven species. (b) Bidirectional replication revealed by deep sequencing at the exponential phase for the strain SAO4-4. Download FIG S5, PDF file, 0.5 MB. Copyright © 2021 Xie et al. 2021 Xie et al. This content is distributed under the terms of the Creative Commons Attribution 4.0 International license.

As shown in the species tree, the available *Pseudoalteromonas* genomes comprised two monophyletic groups, each consisting of two clades (clades I and II versus clades III and IV), with clade I consisting exclusively of the three strains carrying the bidirectionally replicating chromids. With this robust species tree, we applied the parsimonious rule of evolution to infer the evolutionary history of the chromid replication mode. Because a unidirectional replication mode of the chromid at the last common ancestor (LCA) of all *Pseudoalteromonas* requires only one evolutionary change (from uni- to bidirectional replication), whereas a bidirectional mode at the LCA necessitates a minimum of two independent changes (both from bi- to unidirectional replication) to explain the phylogenetic distribution of the chromid replication mode of the extant species, the former was favored (Fig. 4a). Molecular dating analysis suggests that the LCA of *Pseudoalteromonas* arose around 502 to 378 million years ago (MYA) (Fig. S6). Clade I diverged from other lineages 469 to 324 MYA, though the extant *Pseudoalteromonas* members displaying bidirectional replication at the chromid evolved from a more recent ancestor, about 36 to 9 MYA (Fig. S6).

10.1128/mBio.02745-20.6 FIG S6 Estimated divergence time of *Pseudoalteromonas*. Download FIG S6, PDF file, 0.6 MB. Copyright © 2021 Xie et al. 2021 Xie et al. This content is distributed under the terms of the Creative Commons Attribution 4.0 International license.

Colinear analysis (Fig. 4b) suggests that different positions of *ter2* relative to *ori2* in the genus can be explained by gene insertion events between them, resulting in a shift of *ter2* away from *ori2*. Consistently, genome annotation revealed the presence of a number of insertion sequences and integrons in chromids (Fig. 4b, asterisks; Table S4a). Perhaps the most striking feature was that chromids contain long prophage-like regions (57 to 277 kb in total), accounting for 6.5% to 24.1% of the chromid length (Fig. 4b, boxes; Table S4a). Compared to chromids, main chromosomes had much lower ratios of prophage-like regions (1.1% to 6.7%; 36 to 258 kb in length). This result suggests that the insertion of exogenous DNA in chromids occurred at high frequency and that such events may have significantly changed the position of *ter* in the LCA of clade I. Thus, we identified an example showing how insertions of DNA in a chromid led to a switch from unidirectional to bidirectional replication.

10.1128/mBio.02745-20.10 TABLE S4 (a) Integrons, insertion sequences, and prophages predicted on *Pseudoalteromonas* chromids. (b) Homologs of *dif2* sites on chromids (Chr2) of *P. spongiae* JCM12884^T^, *Pseudoalteromonas* sp. SAO4-4, *P. piratica* OCN003^T^, and *P. prydzensis* DSM14232^T^ predicted by BLASTN searches (E value cutoff of 1). Download Table S4, PDF file, 0.7 MB. Copyright © 2021 Xie et al. 2021 Xie et al. This content is distributed under the terms of the Creative Commons Attribution 4.0 International license.

### Replications of the two chromosomes are coordinated to terminate in synchrony.

The appropriate timing of replication is essential for successful cell division (10). Unlike in bacteria with a single chromosome, the presence of multiple chromosomes in a bacterial genome necessitates a cellular mechanism that can coordinate the replication of multiple chromosomes (6). Two conceptual models have been proposed for such coordination: initiation synchrony (control of initiation time) and termination synchrony (control of termination time) (41). Here, we compared *P. tunicata* DSM14096^T^ with *P. spongiae* JCM 12884^T^ to investigate the coordination modes used by unidirectionally versus bidirectionally replicating chromids. Again using deep-sequencing data, we analyzed the sequencing coverage for the main chromosome *ori* and *dif* sites (*ori1* and *dif1*) and the chromid *ori* and *dif* sites (*ori2* and *dif2*). As expected, during the stationary growth phase, a period lacking extensive replication, the sequencing coverage for each *ori* and *dif* site was nearly equal in both species (Fig. 3c). At the exponential phase, however, the *ori1*/*ori2* ratios were 1.5 and 1.8 for *P. spongiae* and *P. tunicata*, respectively, while their *dif1*/*dif2* ratios each were 1.0 (Fig. 3c), indicating that both *Pseudoalteromonas* species we examined adopted termination synchrony to coordinate the replication of the multiple chromosomes in their genomes, despite the fact that they differed in the replication mode (i.e., unidirectional versus bidirectional replications) of their chromids.

### Bidirectionally replicating chromids are larger than unidirectionally replicating chromids.

Yet another striking trend between the bidirectionally and unidirectionally replicating *Pseudoalteromonas* chromids was that the bidirectionally replicating chromids were larger (1.60 to 1.67 Mb versus 0.64 to 1.39 Mb). The coordination of the replication of multiple chromosomes implies that the time required for the complete replication of the main chromosome sets an upper time limit for the chromid replication (10, 42). Compared with unidirectional replication, bidirectional replication needs only half the replication time and therefore allows the chromid size to increase by 1-fold while keeping the same replication time. This was further supported by higher ratios of chromid size to main-chromosome size for bidirectional replication than for unidirectional replication in both the genus *Pseudoalteromonas* (50.6% to 50.8% versus 19.3% to 29.8%) and other bacteria (50.4 ± 25.2% versus 18.8 ± 8.7%) (Fig. 1c). It was also noted that the ratios 50.6% to 50.8% are too high to allow the chromids to finish the replication in unidirectional mode within the bidirectional replication time of the main chromosome. In this scenario, using a bidirectional replication system would allow the replication of larger chromids. The increase in the chromid and thus genome size may increase the metabolic versatility, which is particularly beneficial to copiotrophic bacteria like *Pseudoalteromonas*. Thus, conversion from a unidirectional to a bidirectional replication system may confer a competitive advantage for *Pseudoalteromonas* to explore multiple niches. Consistently, a survey of the publicly available multipartite genomes revealed that 63% of bidirectionally replicating secondary replicons have a size larger than one half of the corresponding main chromosome (Fig. 1c), though other unknown mechanisms may also play roles in the evolution of replication direction.

## DISCUSSION

Bacterial circular chromosomes replicate bidirectionally by the theta mechanism. Circular plasmids replicate by three general mechanisms: theta type, strand displacement, and rolling circle (43). Different machineries are involved in different plasmid replication mechanisms (43). For example, initiation of theta-type plasmid replication generally requires a plasmid-encoded initiator protein. In contrast, initiation of strand displacement replication requires the plasmid-encoded helicase, primase, and initiator protein, and initiation of rolling-circle replication requires the plasmid-encoded protein with DNA strand transferase enzymatic activity. The absence of the above essential proteins for the latter two mechanisms indicated that *Pseudoalteromonas* chromids replicate by the theta mechanism. Consistent with this, no such proteins in chromids from other bacteria have been reported. Considering the different replication factors involved in different mechanisms, a parsimony assumption is that chromids likely evolved from plasmids that also used the theta-type replication. Unlike in chromosomes, however, theta-type replication in plasmids is mostly unidirectional (43). Therefore, it is possible that bidirectionally replicating chromids may have evolved from unidirectionally replicating plasmids. In this situation, the unidirectionally replicating chromids may represent an intermediate stage in the evolutionary trajectory from a unidirectionally replicating plasmid to a bidirectionally replicating chromosome.

Though chromids have been observed in different bacteria, only bidirectionally replicating chromids have been experimentally verified so far (41). Previous bioinformatic analysis predicted that the chromid of P. haloplanktis TAC125 may replicate unidirectionally (19). Here, the existence of unidirectional replication was experimentally verified in *Pseudoalteromonas*. Since a bacterial chromosome typically replicates bidirectionally and is larger than a chromid, the question of whether a small and unidirectionally replicating chromid (more plasmid-like) may evolve into a large and bidirectionally replicating one (more chromosome-like) was raised. Deep genome sequencing and phylogenomic analysis revealed that the last common ancestor of *Pseudoalteromonas* chromids replicated unidirectionally and the unidirectional replication evolved into bidirectional replication only once, occurring at one of the basal clades of the *Pseudoalteromonas* phylogeny. Therefore, profiling of *Pseudoalteromonas* from marine environments ultimately revealed a basic biological mechanism that deepens our understanding of the early evolutionary history of bacterial chromosomes.

In E. coli, the replication fork is blocked by complexes formed by binding of the Tus protein to the *ter* regions (44). The existence of a conserved *tus* gene within the *Pseudoalteromonas* genomes suggests that the genus also employs the Tus/*ter* replication fork trap system. However, with the *ter* consensus sequence of E. coli as the reference (44), a sequence search failed to identify homologues near the expected *ter* region on the *P. tunicata* chromid (i.e., the region with the lowest sequencing coverage as in Fig. 3a), suggesting that the *ter* sequences of *Pseudoalteromonas* are either highly divergent from or evolutionarily unrelated to that of E. coli. Two replication forks are formed at *ori2* on a bidirectionally replicating chromid. It is unclear whether one or two forks are formed on a unidirectionally replicating chromid. In the former situation, the change of replication initiation machinery occurred during the transition from uni- to bidirectional replication to enable the formation of a second replication fork. In the latter, two replication forks are formed at *ori2* on a unidirectionally replicating chromid, and the one proceeding in the counterclockwise direction is immediately blocked at the *ter2* region, which is located very close to the *ori2* site on the unidirectionally replicating chromid. The molecular mechanism of the initiation of unidirectional replication needs further study.

The chromids contained long prophage-like regions that accounted for up to 6.5% to 24.1% of the chromid length, suggesting that insertion of phage genomes may have significantly changed the chromid structure. It was proposed that the insertion events between the *ori2* and *dif2* sites in the unidirectionally replicating ancestor moved *dif2* away from *ori2*, eventually resulting in bidirectionally replicating descendants. Consistently, prophages were annotated at or near the *dif2* site in a few strains (Fig. 4, boxes). Genetic studies of V. cholerae have identified a number of phages and other integrative mobile elements that hijack tyrosine recombinases XerC and XerD from their host for integration (also referred to as IMEXs) (for a review, see reference 45). In most bacteria, XerC and XerD are used to resolve chromosome dimers by binding to *dif* (4, 46). A sequence search with the *dif2* sequence as the query revealed a few *dif2*-like sequences within or near the prophage regions (Table S4b). It was noted that the *dif2* site was located within a prophage in the bidirectionally replicating chromids but not in the unidirectionally replicating chromids (Fig. 4b; see Table S3b for the positions of the *dif2* sites and Table S4a for the positions of the prophages). Prophages were not found between the *dif2* and the *ori2* sites in the unidirectionally replicating chromids either. This observation was consistent with the hypothesis that a few phages and/or other mobile genetic elements might have specifically integrated at the *dif2* site, resulting in the separation of *dif2* from *ori2* as well as the increase of the chromid size.

The switch from uni- to bidirectional replication of the *Pseudoalteromonas* chromids represents a simple strategy that bacteria use to add (a large number of) genes to the chromosome while exerting little influence on the replication physiology of host bacteria. This is because replication initiation, elongation, and termination of chromids are not changed in spite of the fact that hundreds of genes are acquired on the two replichores of a chromid. Provided that the cell harboring the new bidirectionally replicating chromid adopted termination synchrony to coordinate replication, each DNA replication complex would have to replicate only half as much DNA in the allotted replication time period. Thus, there would be more than adequate time for the replication of additional genetic material that was inserted into the now bidirectionally replicating chromid, explaining the trend observed among the *Pseudoalteromonas* genomes that the bidirectionally replicating chromids are larger. While bidirectional replication of the chromid may benefit the organism, the reality is that unidirectional replication is more widespread within the genus. This suggests that other factors may also control the replication mode in this genus, which is a topic of interest for future studies.

The replication of multiple chromosomes within a bacterial cell is well coordinated, and the underlying mechanism has been studied with V. cholerae as a model. The V. cholerae chromid replication is regulated by the initiator RctB encoded by the chromid (Fig. S1c), which binds to iterons (12-mer sites) in *ori2* to promote initiation and binds to 39-mer regulatory sites in *ori2* to strongly inhibit initiation (47). RctB can also bind to a 150-bp site on the main chromosome, an *ori2* replication enhancer, which increases the RctB binding affinity for iterons and decreases affinity for 39-mers in the chromid (48). Replication of this 150-bp site (also named *crtS*) triggers the initiation of chromid replication (10). In spite of the differences in chromid size and mode of replication across the genus *Pseudoalteromonas*, the replication of the two chromosomes in all strains studied was precisely coordinated to terminate in synchrony (Fig. 3 and Fig. S5). This observation suggests that the initiation of chromid replication is controlled by the timing of the main chromosome replication in *Pseudoalteromonas*. Though *Pseudoalteromonas* and V. cholerae use different initiators for chromid replication, it is possible that *Pseudoalteromonas* uses a similar molecular mechanism in the communication between chromosomes, i.e., the replication of a specific site on the main chromosome to trigger the initiation of chromid replication. It was not unexpected that search of the main chromosome sequence of *Pseudoalteromonas* revealed no sites showing similarities to *crtS* carried in V. cholerae, which is probably a result of the different initiators used by the two organisms. The molecular mechanism underlying the synchrony of the replication of multiple chromosomes in *Pseudoalteromonas* is worth further study.

The mode of replication is known to strongly impact the organization of bacterial chromosomes (11, 49–51). The replication mode leads to the base composition asymmetries on the chromosome, and one such asymmetry, GC skew, has been used to predict the position of the *ori* site (52). Here, experimental verification of the existence of unidirectional replication in the *Pseudoalteromonas* chromid suggests that the GC skew is a valuable predictor of the replication mode of bacterial chromosomes. Other demonstrated replication-dependent structural features include unequal distributions of essential genes between the two replication strands, a propensity for highly expressed genes to be clustered near the *ori* site, and the accumulation of recombination-associated *chi* sites in the leading strand (50). The current study presents an example of the opposite situation wherein a structural change shifts the mode of replication used by a chromosome. Furthermore, our study revealed a higher number of genes on the leading strand than the lagging strand for both replichores of the *Pseudoalteromonas* main chromosomes (leading-lagging ratios of 1.61 ± 0.07 and 1.53 ± 0.07 for the right and left replichores, respectively; *n* = 9). Similarly, there were more genes on the leading strand for the unidirectionally replicating chromids (1.34 ± 0.10; *n* = 21) and for the right replichores of the bidirectionally replicating chromids (1.26 ± 0.13; *n* = 3). However, such unequal distribution of genes on the two strands was not apparent on the left replichore of the bidirectionally replicating chromids (1.04 ± 0.02; *n* = 3), consistent with the hypothesis that the left replichore was newly generated from exogenous DNA insertion between the *ori2* and *dif2* sites. Replication also has a global impact on the mutation rate. Generally, there are higher mutation rates in late-replicating regions (14), and the secondary chromosome shows higher mutation rates than the main chromosome (15). Recent studies of genomes possessing one single chromosome and multiple chromosomes revealed that the correlation between the mutation rate and replication timing is not simple but periodic (12, 13, 53–56). The genus *Pseudoalteromonas* contains both unidirectionally and bidirectionally replicating members, representing a new model to study the mutation rate variation across chromosome.

With the marine bacterial genus *Pseudoalteromonas* as the model, our study experimentally demonstrated the existence of unidirectional replication in bacterial chromosomes and revealed both uni- and bidirectional replication modes in the homologous chromids from different strains. Our results suggest that the uni- and bidirectionally replicating chromids represent two stages in the evolutionary history from a unidirectionally replicating plasmid to a bidirectionally replicating chromosome. Our bioinformatic analyses of unrelated bacterial lineages predicted that, while most additional large replicons replicate bidirectionally, a few may also replicate unidirectionally, suggesting that the evolution from unidirectionally replicating plasmids to chromosomes (chromids) may have occurred multiple times in bacteria.

## MATERIALS AND METHODS

### Genome sequencing and assembly.

Type strains for 26 *Pseudoalteromonas* species were purchased from the Leibniz Institute DSMZ-German Collection of Microorganisms and Cell Cultures (strains 14585, 9414, 6820, 17587, 16473, 18437, 26439, 14402, 8810, 6065, 15925, 9166, 14232, 15203, 6057, 8771, 6061, 6842, 14001, 14401, 15557, and 14096) and the Japan Collection of Microorganisms (strains 15903, 21460, 20779, and 12884). The complete list of strains is presented in Table S2.

Genomic DNA was extracted using the PowerMax soil DNA isolation kit (Mo Bio laboratories, Inc., USA) according to the manufacturer’s instructions. Multiple Illumina paired-end DNA libraries of different insert sizes (500 bp, 2 kbp, 5 kbp, and 10 kbp) were prepared for each strain (Table S2). Genome sequencing was performed using the Illumina HiSeq 2000 platform. The read length was 90 bp. Clean reads were obtained by removing those containing ≥36-bp low-quality bases (Phred score, ≤20), those containing ≥9 Ns, and those containing adapter contamination with FASTX-Toolkit version 0.0.13 (http://hannonlab.cshl.edu/fastx_toolkit/). The resultant clean reads were assembled using SOAPdenovo version 1.05 (57), with reads from the 500-bp paired-end library being used to create contigs and reads from 2-kb, 5-kb, and 10-kb libraries used to construct scaffolds (pair_num_cutoff = 5, map_len = 80) (Table S2). A few intrascaffold gaps were closed using reads from the 500-bp paired-end library by GapCloser version 1.1 (http://soap.genomics.org.cn/soapdenovo.html). The remaining intra- and interscaffold gaps were closed using PCR. For the intrascaffold gaps, PCR primer pairs were designed on the flanking contig ends. To design primer pairs for closing the interscaffold gaps, the linkage information between scaffolds was predicted by comparison with genomes of strains TAC125 and SM9913 as well as other species sequenced in this study, using MUMmer version 3.23 (58). Sequences of PCR products were determined by directed sequencing (primer walking). Scaffolds were split into contigs at unclosed gaps. The resultant assemblies were used for further annotation. Detailed information on sequencing and assembly is available in Table S2.

Open reading frames were predicted using Glimmer version 3.02 (59). Gene functions were annotated using both the NCBI nonredundant protein database and the Cluster of Orthologous Groups of proteins (COG) database (60) using BLASTP (E value cutoff, 1e−5; score cutoff, 60; coverage cutoff, 50% for both query and target; and identity cutoff, 35%).

### GC skew analysis.

The GC skew is calculated as (number of Gs – number of Cs)/(number of Gs + number of Cs), with a sliding window. The cumulative GC skew was calculated by summing up the GC skew values for all windows from the start window to the current window along the replicon. The circular representation of GC skew was produced using DNAPlotter (61).

To explore the replication direction of bacterial chromosomes, all complete bacterial genomes in the NCBI RefSeq database (May 2019 version) were downloaded. Then, GC skew for all large replicons (>200 kb) was calculated using a self-written Perl script, with a window size of 1 kb and a step size of 1 kb. Lastly, the GC skew and cumulative GC skew were manually inspected. The chromosomes of E. coli, B. subtilis, and V. cholerae were used as standards for bidirectional replication. The chromid of P. haloplanktis TAC125 was used as a standard for unidirectional replication.

### *In silico* analysis of *ori* and *dif* sites.

The *ori* sites were annotated based on the sequence similarities with the reference sequences, the locus of the neighboring gene *dnaA* (for *ori1*) or *repA* (for *ori2*), and the internal structures, including DnaA boxes and Dam methylation sites (52). With the *ori* sites (*ori1* and *ori2*) of TAC125, V. cholerae, and E. coli as references, genome sequences were searched using BLASTN with an E value cutoff of 1e−5, a score cutoff of 60, a coverage cutoff of 50% (for both query and target), and an identity cutoff of 30%. The 28-bp *dif* sites were searched with *dif* sites of TAC125, V. cholerae, and E. coli as references using BLASTN with an E value cutoff of 1 and a query coverage cutoff of 40%. The obtained candidate *dif* sites were further confirmed based on the following criteria: located in noncoding sequences and roughly opposite or near the *ori* site and possessing a palindromic structure and a highly conserved XerD binding site (62).

### Characterization of the minimal replicon.

The minimal replicon for the chromid was characterized by testing the ability of the *ori2* site and its flanking sequences to initiate the replication of the vector pOriT-4CM in *Pseudoalteromonas*. pOriT-4CM is highly similar to the shuttle vector pOriT-4Em, which can replicate in both E. coli and *Pseudoalteromonas* (63). The only difference between the two vectors is that pOriT-4Em contains an erythromycin resistance (Em^r^) gene, while pOriT-4CM contains a chloramphenicol resistance (Cm^r^) gene (see Fig. S3a for map of pOriT-4CM). First, the vector pOriT-4CM was digested with BamHI and PstI to delete the sequence for replication initiation in *Pseudoalteromonas*. Next, a set of sequences including the *ori2* site and the flanking sequences from the chromid were amplified by PCR and were then ligated into the linearized pOriT-4CM by using an In-Fusion HD Plus cloning kit (TaKaRa, Japan) to obtain a series of recombinant plasmids (p4CM-pspo-1, p4CM-pspo-2, p4CM-pspo-3, p4CM-pspo-4, p4CM-ptun-1, p4CM-ptun-2, p4CM-ptun-3, and p4CM-ptun-4). These plasmids were transformed into E. coli DH5α cells (Trans, China) and then were isolated and verified by Sanger DNA sequencing. The verified plasmids were then introduced into conjugation donor strain E. coli WM3064. Next, the plasmids were transferred into *P. spongiae* JCM 12884^T^ or *P. tunicata* DSM14096^T^ by intergeneric conjugations, as described previously (64). *P. spongiae* and *P. tunicata* were grown on marine LB agar medium with 100 μg/ml ampicillin and 35 μg/ml chloramphenicol (Sangon Biotech, China) at 25°C to screen single colonies that contain the recombinant plasmids. Each single colony was then grown in marine LB broth supplemented with 100 μg/ml ampicillin and 35 μg/ml chloramphenicol, and the recombinant plasmids were extracted and verified by restriction enzyme digestion. The bacterial strains and plasmids used in this study are listed in Fig. S3d.

### Deep genome sequencing and chromosome coverage analysis.

For each strain, synchronized cells were obtained by starving in artificial seawater at 25°C for 24 h as described previously (41). These cells were then cultivated in marine broth 2216 at 25°C and harvested at the exponential phase (1 to 4 h) and the stationary phase (38 to 40 h). Genomic DNA was extracted using the PowerMax soil DNA isolation kit (Mo Bio laboratories, Inc., USA) according to the product instructions and sequenced with an Illumina HiSeq 2500 system (Illumina, USA). A 300-bp insert size library was constructed for each sample. Clean reads were mapped to replicons using Burrows-Wheeler Aligner (BWA) (65). The coverage of every base pair was calculated using the mpileup subprogram in SAMtools v.1.3.1 (66). The coverage data were further grouped in bins of 1,000 bp. For *P. tunicata* and *P. spongiae*, the potential bias in the exponential-phase coverage was removed by correction using the stationary-phase coverage. First, based on the stationary-phase data, a correction factor for each bin was calculated as the coverage of the bin divided by the average coverage of the replicon. Next, the exponential-phase coverage of each bin was corrected after division by the corresponding correction factor. Besides, outliers were removed from the coverage profiles by comparing the coverage of each bin with its 50 neighboring bins and removing bins with a coverage difference that exceeded twice the difference between the first quartile and the third quartile of coverages of neighboring bins.

### Phylogenomic analysis.

Orthologous genes were found by using OrthoFinder v2.2.1 (67). Single-copy genes were selected for the main chromosome and the chromid, respectively. Amino acid sequences were aligned with MAFFT v7.222 with default parameters (68). Alignments were trimmed using trimAl v1.4 with the parameter –st 0.001 (69). We also removed compositionally heterogeneous genes which may affect phylogenetic inference using P4 v1.2.0 (70) with the simulation-based test at a significance level of 0.05. Then, the concatenated alignments were used to reconstruct a maximum-likelihood tree using IQ-TREE v1.6.11 (71), with the model LG+G+F+C60 under the posterior mean site frequency (PMSF) approximation (72). This comprehensive model uses site-specific amino acid profiles based upon the C60 empirical frequency profiles (73). This model has been increasingly recommended for phylogenomic analysis of species that diverged very long ago and can also largely reduce the impact of long branch attraction (72, 74, 75). For the main chromosome tree, Algicola sagamiensis DSM 14643, a close relative of *Pseudoalteromonas*, was used as the outgroup. For the chromid tree, the tree was rooted at the midpoint, since the close relatives of the genus *Pseudoalteromonas* have no chromids and therefore cannot be used as the outgroup. Comparisons between different topologies were performed using the seven tests implemented in IQ-Tree (Fig. S2b).

### Molecular dating of the divergence time of the *Pseudoalteromonas*.

In order to estimate the time of transition from unidirectional replication to bidirectional replication, molecular dating analysis was performed using MCMCTree (76) with the relaxed-clock model and approximate likelihood calculation (77) on the basis of amino acid alignments of 25 conserved bacterial proteins used in the study (78). Outgroup species were selected based on the study (79) and four calibration points were used, which correspond to the split time of alpha- and gammaproteobacteria, the origin of cyanobacteria, the origin of the *Pleurocapsales*, and the origin of the *Nostocales*, respectively (nodes 1 to 4 in Fig. S6). The age of node 1 was set to a range from 2,620 to 2,360 million years ago (MYA) (80), and the age of node 2 was set to range from 3,000 to 2,320 MYA based on geochemical evidence for the rise of oxygen (81, 82). The age ranges of node 3 and node 4 were set to be 1,900 to 1,700 MYA and 1,900 to 1,600 MYA, respectively, based on a previous study (83). Alternatively, the age ranges of node 3 and node 4 were also set to be 2,450 to 1,750 MYA and 2,450 to 2,100 MYA, respectively, based on a previous study (84), and a similar divergence time was predicted for the emergence of bidirectional replication.

### Annotation of insertion sequences on chromids.

Insertion sequences were annotated using the ISsaga server with default parameters (http://issaga.biotoul.fr/issaga_index.php) (85). Integrons and cassette arrays were annotated using the IntegronFinder server (86). Prophage loci were annotated using phiSpy v2.1 with default parameters (87) and the PHASTER server (88).

### Data availability.

Genome sequences obtained in this study have been deposited in GenBank/DDBJ under accession numbers CP011011, CP011012, CP011025 to CP011042, CP011924, CP011925, AHBZ00000000, AHCB00000000, AHCD00000000, AHCF00000000, AQGU00000000 to AQGW00000000, AQGY00000000, AQHA00000000 to AQHC00000000, AQHE00000000 to AQHH00000000, and AQHJ00000000. The alignments, phylogenies, and scripts (89) are available at https://figshare.com/s/2145896d75319c896d58.
