# Supplementary material for: Evolutionary Trajectory of the Replication Mode of Bacterial Replicons
Source: mBio. 2021 Jan 26;12(1):e02745-20. doi: 10.1128/mBio.02745-20 (PMC7858055; doi:10.1128/mBio.02745-20)
Supplement: FIG S1 [file mBio.02745-20-sf001.pdf]

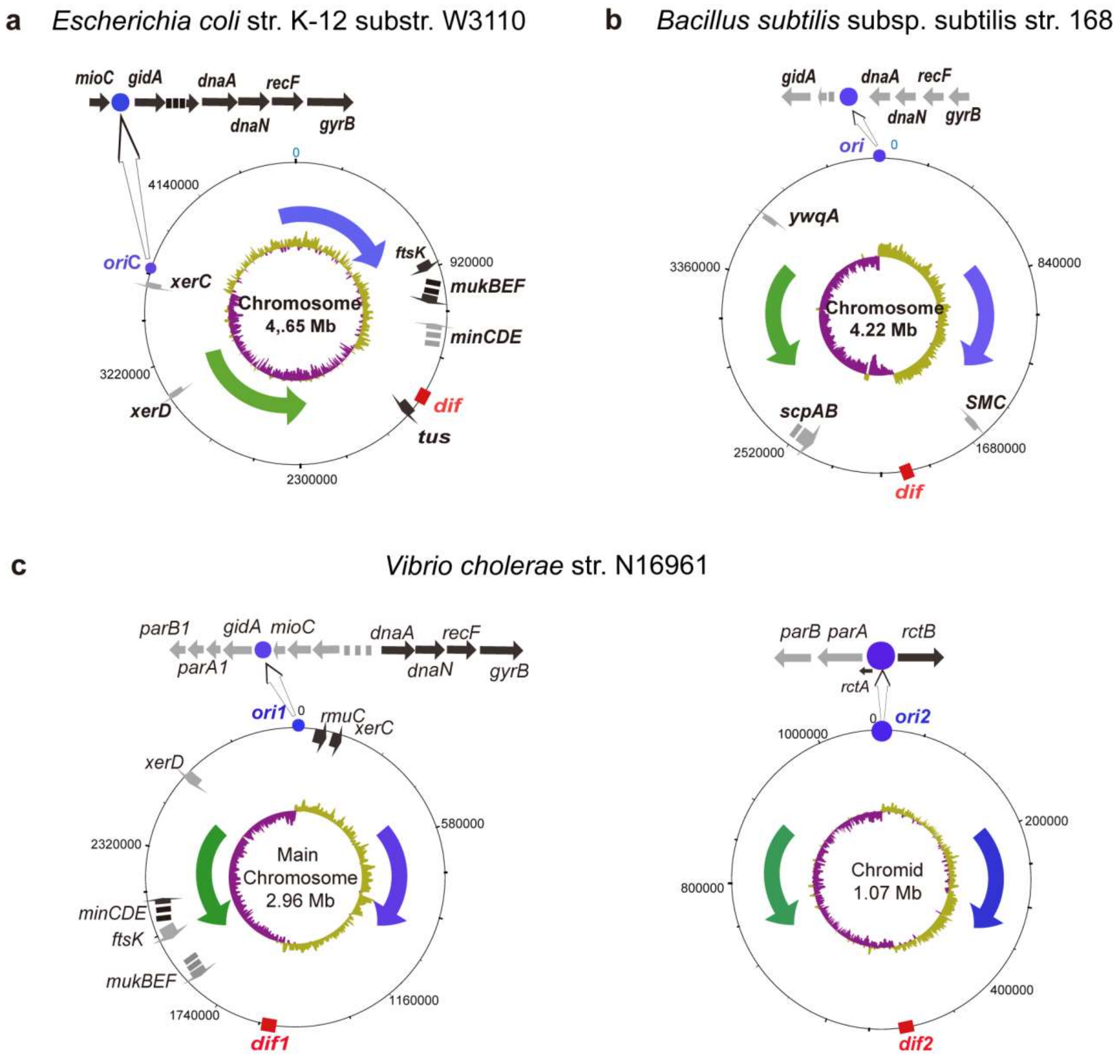

Supplementary Figure S1. The chromosome structure for model bacteria *Escherichia coli* (a), *Bacillus subtilis* (b), and *Vibrio cholerae* (c). The *ori* site (blue solid circle), *dif* site (red solid rectangle), and genes related to main chromosome/chromid replication and maintenance are indicated on the outer circle. GC skew is shown as inner circle. Replication directions are shown as blue (clockwise) and green (counter clockwise) arrows.
