# Supplementary material for: Evolutionary Trajectory of the Replication Mode of Bacterial Replicons
Source: mBio. 2021 Jan 26;12(1):e02745-20. doi: 10.1128/mBio.02745-20 (PMC7858055; doi:10.1128/mBio.02745-20)
Supplement: TABLE S1 [file mBio.02745-20-st001.pdf]

Table S1. Predicted unidirectionally replicating replicons (> 200 kb) from NCBI RefSeq database based on GC skew..

Strains	GenBank acc.	Replicon name	Size (bp)	Taxonomy rank
Stenotrophomona s sp. KCTC 12332	NZ_CP014274.1	main chromosome	4541594	Bacteria; Proteobacteria; Gammaproteobacteria; Xanthomonadales; Xanthomonadaceae; Stenotrophomonas.
Phreatobacter cathodiphilus	NZ_CP027668.1	main chromosome	4494445	Bacteria; Proteobacteria; Alphaproteobacteria; Phreatobacter.
Devosia sp. I507	NZ_CP026747.1	main chromosome	4005916	Bacteria; Proteobacteria; Alphaproteobacteria; Rhizobiales; Hyphomicrobiaceae; Devosia.
Halorhodospira halophila SL1	NC_008789.1	main chromosome	2678452	Bacteria; Proteobacteria; Gammaproteobacteria; Chromatiales; Ectothiorhodospiraceae; Halorhodospira.
Pseudoalteromonas sp. R3	NZ_CP034834.1	chromosome 2	1328532	Bacteria; Proteobacteria; Gammaproteobacteria; Alteromonadales; Pseudoalteromonadaceae; Pseudoalteromonas.
Pseudoalteromonas luteoviolacea	NZ_CP015414.1	chromosome 2	1321127	Bacteria; Proteobacteria; Gammaproteobacteria; Alteromonadales; Pseudoalteromonadaceae; Pseudoalteromonas.
Pseudoalteromonas luteoviolacea	NZ_CP015412.1	chromosome 2	1321096	Bacteria; Proteobacteria; Gammaproteobacteria; Alteromonadales; Pseudoalteromonadaceae; Pseudoalteromonas.
Pseudoalteromonas luteoviolacea	NZ_CP015416.1	chromosome 2	1321025	Bacteria; Proteobacteria; Gammaproteobacteria; Alteromonadales; Pseudoalteromonadaceae; Pseudoalteromonas.
Pseudoalteromonas rubra	NZ_CP013612.1	chromosome 2	1311023	Bacteria; Proteobacteria; Gammaproteobacteria; Alteromonadales; Pseudoalteromonadaceae; Pseudoalteromonas.
Agrobacterium vitis S4	NC_011988.1	chromosome 2	1283187	Bacteria; Proteobacteria; Alphaproteobacteria; Rhizobiales; Rhizobiaceae; Rhizobium/Agrobacterium group; Agrobacterium.
Pseudoalteromonas piscicida	NZ_CP031760.1	chromosome 2	1261888	Bacteria; Proteobacteria; Gammaproteobacteria; Alteromonadales; Pseudoalteromonadaceae; Pseudoalteromonas.
Pseudoalteromonas piscicida	NZ_CP021647.1	chromosome 2	1188838	Bacteria; Proteobacteria; Gammaproteobacteria; Alteromonadales; Pseudoalteromonadaceae; Pseudoalteromonas.
Pseudoalteromonas piscicida	NZ_CP031762.1	chromosome 2	1188822	Bacteria; Proteobacteria; Gammaproteobacteria; Alteromonadales; Pseudoalteromonadaceae; Pseudoalteromonas.
Pseudoalteromonas phenolica	NZ_CP013188.1	chromosome 2	1023473	Bacteria; Proteobacteria; Gammaproteobacteria; Alteromonadales; Pseudoalteromonadaceae; Pseudoalteromonas.
Pseudoalteromonas tunicata	NZ_CP031962.1	chromosome 2	982384	Bacteria; Proteobacteria; Gammaproteobacteria; Alteromonadales; Pseudoalteromonadaceae; Pseudoalteromonas.
Pseudoalteromonas tunicata	NZ_CP011033.1	chromosome 2	980475	Bacteria; Proteobacteria; Gammaproteobacteria; Alteromonadales; Pseudoalteromonadaceae; Pseudoalteromonas.
Pseudoalteromonas sp. 1_2015MBL_MicDiv	NZ_CP012738.1	chromosome 2	907248	Bacteria; Proteobacteria; Gammaproteobacteria; Alteromonadales; Pseudoalteromonadaceae; Pseudoalteromonas.
Pseudoalteromonas sp. Bsw20308	NZ_CP013139.1	plasmid pPBSW1	906103	Bacteria; Proteobacteria; Gammaproteobacteria; Alteromonadales; Pseudoalteromonadaceae; Pseudoalteromonas.
Pseudoalteromonas agarivorans DSM 14585	NZ_CP011012.1	chromosome 2	843022	Bacteria; Proteobacteria; Gammaproteobacteria; Alteromonadales; Pseudoalteromonadaceae; Pseudoalteromonas.
Pseudoalteromonas donghaensis	NZ_CP032091.1	plasmid unnamed1	842855	Bacteria; Proteobacteria; Gammaproteobacteria; Alteromonadales; Pseudoalteromonadaceae; Pseudoalteromonas.
Pseudoalteromonas sp. Xi13	NZ_CP034440.1	chromosome 2	837013	Bacteria; Proteobacteria; Gammaproteobacteria; Alteromonadales; Pseudoalteromonadaceae; Pseudoalteromonas.
Pseudoalteromonas agarivorans	NZ_CP033066.1	chromosome 2	824720	Bacteria; Proteobacteria; Gammaproteobacteria; Alteromonadales; Pseudoalteromonadaceae; Pseudoalteromonas.
Pseudoalteromonas carrageenovora	NZ_CP027524.1	chromosome 2	820457	Bacteria; Proteobacteria; Gammaproteobacteria; Alteromonadales; Pseudoalteromonadaceae; Pseudoalteromonas.
Listeria monocytogenes	NZ_LR134401.1	plasmid: 4	809616	Bacteria; Firmicutes; Bacilli; Bacillales; Listeriaceae; Listeria.
Pseudoalteromonas arctica A 37-1-2	NZ_CP011026.1	chromosome 2	783876	Bacteria; Proteobacteria; Gammaproteobacteria; Alteromonadales; Pseudoalteromonadaceae; Pseudoalteromonas.
Pseudoalteromonas espejiana DSM 9414	NZ_CP011029.1	chromosome 2	779695	Bacteria; Proteobacteria; Gammaproteobacteria; Alteromonadales; Pseudoalteromonadaceae; Pseudoalteromonas.
Neorhizobium sp. NCHU2750	NZ_CP030828.1	plasmid pNCHU2750a	764863	Bacteria; Proteobacteria; Alphaproteobacteria; Rhizobiales; Rhizobiaceae; Rhizobium/Agrobacterium group; Neorhizobium.
Pseudoalteromonas translucida KMM 520	NZ_CP011035.1	chromosome 2	757205	Bacteria; Proteobacteria; Gammaproteobacteria; Alteromonadales; Pseudoalteromonadaceae; Pseudoalteromonas.
Pseudoalteromonas sp. 13-15	NZ_CP019163.1	chromosome 2	740378	Bacteria; Proteobacteria; Gammaproteobacteria; Alteromonadales; Pseudoalteromonadaceae; Pseudoalteromonas.
Pseudoalteromonas tetraodonis	NZ_CP011042.1	chromosome 2	729257	Bacteria; Proteobacteria; Gammaproteobacteria; Alteromonadales; Pseudoalteromonadaceae; Pseudoalteromonas.
Pseudoalteromonas issachenkonii	NZ_CP013351.1	chromosome 2	729217	Bacteria; Proteobacteria; Gammaproteobacteria; Alteromonadales; Pseudoalteromonadaceae; Pseudoalteromonas.
Pseudoalteromonas issachenkonii	NZ_CP011031.1	chromosome 2	728958	Bacteria; Proteobacteria; Gammaproteobacteria; Alteromonadales; Pseudoalteromonadaceae; Pseudoalteromonas.
Salmonella enterica subsp. enterica serovar Senftenberg	NZ_LN868944.1	plasmid : 2	727905	Bacteria; Proteobacteria; Gammaproteobacteria; Enterobacterales; Enterobacteriaceae; Salmonella.
Prevotella sp. oral taxon 299 str. F0039	NC_022124.1	main chromosome	709850	Bacteria; Bacteroidetes; Bacteroidia; Bacteroidales; Prevotellaceae; Prevotella.
Bosea sp. RAC05	NZ_CP016463.1	plasmid pBSY19_1	708738	Bacteria; Proteobacteria; Alphaproteobacteria; Rhizobiales; Bradyrhizobiaceae; Bosea.
Pseudoalteromonas sp. SM9913	NC_014800.1	chromosome 2	704884	Bacteria; Proteobacteria; Gammaproteobacteria; Alteromonadales; Pseudoalteromonadaceae; Pseudoalteromonas.
Anderseniella sp. Alg231-50	NZ_LT703004.1	chromosome 2	704593	Bacteria; Proteobacteria; Alphaproteobacteria; Rhizobiales; Rhodobiaceae; Anderseniella.
Pseudoalteromonas nigrifaciens	NZ_CP011037.1	chromosome 2	679846	Bacteria; Proteobacteria; Gammaproteobacteria; Alteromonadales; Pseudoalteromonadaceae; Pseudoalteromonas.
Pseudoalteromonas sp. DL-6	NZ_CP019771.1	chromosome 2	664854	Bacteria; Proteobacteria; Gammaproteobacteria; Alteromonadales; Pseudoalteromonadaceae; Pseudoalteromonas.
Acinetobacter baumannii	NZ_CP040048.1	plasmid unnamed1	657699	Bacteria; Proteobacteria; Gammaproteobacteria; Pseudomonadales; Moraxellaceae; Acinetobacter; Acinetobacter calcoaceticus/baumannii complex.
Pseudoalteromonas haloplanktis TAC125	NC_007482.1	chromosome 2	635328	Bacteria; Proteobacteria; Gammaproteobacteria; Alteromonadales; Pseudoalteromonadaceae; Pseudoalteromonas.
Alteromonas mediterranea	NZ_CP018028.1	plasmid MCP49-600	610127	Bacteria; Proteobacteria; Gammaproteobacteria; Alteromonadales; Alteromonadaceae; Alteromonas.
Alteromonas mediterranea	NZ_CP018025.1	plasmid pAMCP48-600	603655	Bacteria; Proteobacteria; Gammaproteobacteria; Alteromonadales; Alteromonadaceae; Alteromonas.
Listeria monocytogenes	NZ_LR134400.1	plasmid: 3	593685	Bacteria; Firmicutes; Bacilli; Bacillales; Listeriaceae; Listeria.
Listeria monocytogenes	NZ_LR134402.1	plasmid: 5	568990	Bacteria; Firmicutes; Bacilli; Bacillales; Listeriaceae; Listeria.
Acinetobacter baumannii	NZ_CP040041.1	plasmid unnamed1	561419	Bacteria; Proteobacteria; Gammaproteobacteria; Pseudomonadales; Moraxellaceae; Acinetobacter; Acinetobacter calcoaceticus/baumannii complex.
Xanthomonas sacchari	NZ_CP010410.1	plasmid	508653	Bacteria; Proteobacteria; Gammaproteobacteria; Xanthomonadales; Xanthomonadaceae; Xanthomonas.
Erythrobacter sp. YH-07	NZ_CP031358.1	plasmid unnamed	407975	Bacteria; Proteobacteria; Alphaproteobacteria; Sphingomonadales; Erythrobacteraceae; Erythrobacter.
Rhizobium leguminosarum bv. viciae	NZ_CP022567.1	plasmid pSK03	407945	Bacteria; Proteobacteria; Alphaproteobacteria; Rhizobiales; Rhizobiaceae; Rhizobium/Agrobacterium group; Rhizobium.
Listeria monocytogenes	NZ_LR134403.1	plasmid: 6	407263	Bacteria; Firmicutes; Bacilli; Bacillales; Listeriaceae; Listeria.
Labrenzia aggregata	NZ_CP019631.1	plasmid unnamed1	407152	Bacteria; Proteobacteria; Alphaproteobacteria; Rhodobacterales; Rhodobacteraceae; Labrenzia.
Anderseniella sp. Alg231-50	NZ_LT703005.1	chromosome 3	406731	Bacteria; Proteobacteria; Alphaproteobacteria; Rhizobiales; Rhodobiaceae; Anderseniella.
Anderseniella sp. Alg231-50	NZ_LT703006.1	chromosome 4	399180	Bacteria; Proteobacteria; Alphaproteobacteria; Rhizobiales; Rhodobiaceae; Anderseniella.
Listeria monocytogenes	NZ_LR134399.1	plasmid: 2	393943	Bacteria; Firmicutes; Bacilli; Bacillales; Listeriaceae; Listeria.
Fusobacterium nucleatum *subsp. vincentii 3_1_27*	NZ_CP007065.1	chromosome 2	372621	Bacteria; Fusobacteria; Fusobacteriia; Fusobacteriales; Fusobacteriaceae; Fusobacterium.
Pseudomonas frederiksbergensis	NZ_CP017887.1	plasmid unnamed1	371069	Bacteria; Proteobacteria; Gammaproteobacteria; Pseudomonadales; Pseudomonadaceae; Pseudomonas.
Anderseniella sp. Alg231-50	NZ_LT703007.1	chromosome 5	365660	Bacteria; Proteobacteria; Alphaproteobacteria; Rhizobiales; Rhodobiaceae; Anderseniella.
Butyrivibrio proteoclasticus B316	NC_014389.1	plasmid pCY360	361399	Bacteria; Firmicutes; Clostridia; Clostridiales; Lachnospiraceae; Butyrivibrio.
Pannonibacter phragmitetus	NZ_CP013069.1	plasmid p.p-1	351005	Bacteria; Proteobacteria; Alphaproteobacteria; Rhodobacterales; Rhodobacteraceae; Pannonibacter.
Pannonibacter phragmitetus BB	NZ_CP032313.1	plasmid p.BB_1	349969	Bacteria; Proteobacteria; Alphaproteobacteria; Rhodobacterales; Rhodobacteraceae; Pannonibacter.
Bacillus thuringiensis	NZ_CP039723.1	plasmid p2	349603	Bacteria; Firmicutes; Bacilli; Bacillales; Bacillaceae; Bacillus; Bacillus cereus group.
Bacillus thuringiensis HD1002	NZ_CP009348.1	plasmid 2	349602	Bacteria; Firmicutes; Bacilli; Bacillales; Bacillaceae; Bacillus; Bacillus cereus group.

Bacillus thuringiensis	NZ_CP009334.1	plasmid 2	349601	Bacteria; Firmicutes; Bacilli; Bacillales; Bacillaceae; Bacillus; Bacillus cereus group.
Bacillus thuringiensis HD-789	NC_018516.1	plasmid pBTHD789-1	349599	Bacteria; Firmicutes; Bacilli; Bacillales; Bacillaceae; Bacillus; Bacillus cereus group.
Sphingosinicella sp. BN140058	NZ_CP035502.1	plasmid p1	347485	Bacteria; Proteobacteria; Alphaproteobacteria; Sphingomonadales; Sphingomonadaceae; Sphingosinicella.
Vibrio breoganii	NZ_CP016179.1	plasmid unnamed1	340942	Bacteria; Proteobacteria; Gammaproteobacteria; Vibrionales; Vibrionaceae; Vibrio.
Deinococcus ficus	NZ_CP021084.1	plasmid pDFI3	311736	Bacteria; Deinococcus-Thermus; Deinococci; Deinococcales; Deinococcaceae; Deinococcus.
Alteromonas mediterranea	NZ_CP018030.1	plasmid MRG65-300	302350	Bacteria; Proteobacteria; Gammaproteobacteria; Alteromonadales; Alteromonadaceae; Alteromonas.
Phyllobacterium zundukense	NZ_CP017944.1	plasmid unnamed4	286438	Bacteria; Proteobacteria; Alphaproteobacteria; Rhizobiales; Phyllobacteriaceae; Phyllobacterium.
Clostridium botulinum CDC_1436	NZ_CP006909.1	plasmid pCBG	275986	Bacteria; Firmicutes; Clostridia; Clostridiales; Clostridiaceae; Clostridium.
Clostridium botulinum	NZ_CP031095.1	plasmid p1_CDC51232	270024	Bacteria; Firmicutes; Clostridia; Clostridiales; Clostridiaceae; Clostridium.
Clostridium botulinum Ba4 str. 657	NC_012654.1	plasmid pCLJ	270022	Bacteria; Firmicutes; Clostridia; Clostridiales; Clostridiaceae; Clostridium.
Clostridium botulinum	NZ_CP013700.1	plasmid pRSJ11_1	267642	Bacteria; Firmicutes; Clostridia; Clostridiales; Clostridiaceae; Clostridium.
Clostridium botulinum	NZ_CP013684.1	plasmid pRSJ10_1	266230	Bacteria; Firmicutes; Clostridia; Clostridiales; Clostridiaceae; Clostridium.
Anoxybacillus amylolyticus	NZ_CP015439.1	plasmid pDSM15939_1	258230	Bacteria; Firmicutes; Bacilli; Bacillales; Bacillaceae; Anoxybacillus.
Erythrobacter gangjinensis	NZ_CP018098.1	chromosome 2	229922	Bacteria; Proteobacteria; Alphaproteobacteria; Sphingomonadales; Erythrobacteraceae; Erythrobacter.
Catenovulum sp. CCB-QB4	NZ_CP026605.1	plasmid unnamed1	208085	Bacteria; Proteobacteria; Gammaproteobacteria; Alteromonadales; Alteromonadaceae; Catenovulum.
