# Supplementary material for: Evolutionary Trajectory of the Replication Mode of Bacterial Replicons
Source: mBio. 2021 Jan 26;12(1):e02745-20. doi: 10.1128/mBio.02745-20 (PMC7858055; doi:10.1128/mBio.02745-20)
Supplement: TABLE S2 [file mBio.02745-20-st002.pdf]

**Table S2. Detailed information for 26 *Pseudoalteromonas* type strains sequenced in this study.**

| Spec. (Abbr.)* | Strain | Source | Pigmentation | Paired-end library data (Mb) 500-bp | 2-kb | 5-kb | 10-kb | Total | Cov. | SOAPdenovo K-mer** | PCR gap closing Num | Len (bp) | Contig Num | Total len (bp) | Max (bp) | N50 (bp) | N90 (bp) | GC% | tRNA | *rrn* | Status | ORFs | Chr 2 Status | Ctg. | Size (bp) | Chr2/1 (%) | Accession |
|---|---|---|---|---|---|---|---|---|---|---|---|---|---|---|---|---|---|---|---|---|---|---|---|---|---|---|---|
| ***P. agarivorans (Paga)*** | DSM 14585$^T$ | ascidians | no | 510 | 250 | 250 | | 1010 | 222x | 69 | 11 | 60135 | 2 | 4544962 | 3701940 | 3701940 | 843022 | 40.8 | 104 | 8 | Compl. | 4168 | Compl. | 1 | 843022 | 22.8 | CP011011, CP011012 |
| ***P. arctica (Parc)*** | DSM 18437$^T$ | seawater | no | 520 | 250 | 250 | | 1020 | 216x | 67 | 25 | 56613 | 3 | 4721971 | 3840834 | 3840834 | 783876 | 39.2 | 104 | 9 | Compl. | 4297 | Compl. | 1 | 783876 | 20.4 | CP011025 - CP011027 |
| ***P. espejiana (Pesp)*** | DSM 9414$^T$ | seawater | no | 510 | 250 | 250 | | 1010 | 224x | 61 | 25 | 56067 | 2 | 4500451 | 3720756 | 3720756 | 779695 | 40.3 | 101 | 8 | Compl. | 4061 | Compl. | 1 | 779695 | 21.0 | CP011028, CP011029 |
| ***P. issachenkonii (Piss)*** | DSM 15925$^T$ | brown alga | no | 500 | | 250 | | 750 | 183x | 61 | 12 | 43221 | 2 | 4132618 | 3403660 | 3403660 | 728958 | 40.3 | 103 | 8 | Compl. | 3774 | Compl. | 1 | 728958 | 21.4 | CP011030, CP011031 |
| ***P. nigrifaciens (Pnig)*** | DSM 8810$^T$ | butter | melanin | 500 | | 250 | | 750 | 175x | 61 | 26 | 58838 | 3 | 4274777 | 3521200 | 3521200 | 679846 | 40.2 | 106 | 9 | Compl. | 3996 | Compl. | 1 | 679846 | 19.3 | CP011036 - CP011038 |
| ***P. spongiae (Pspo)*** | JCM 12884$^T$ | sponge | yes | 519 | 250 | 250 | | 1019 | 215x | 61 | 22 | 46884 | 2 | 4752413 | 3154175 | 3154175 | 1598238 | 40.9 | 93 | 7 | Compl. | 4346 | Compl. | 1 | 1598238 | 50.7 | CP011039, CP011040 |
| ***P. tetraodonis (Ptet)*** | DSM 9166$^T$ | puffer fish | no | 510 | 250 | 250 | | 1010 | 245x | 63 | 24 | 53666 | 2 | 4128400 | 3399143 | 3399143 | 729257 | 40.3 | 117 | 8 | Compl. | 3791 | Compl. | 1 | 729257 | 21.5 | CP011041, CP011042 |
| ***P. translucida (Ptra)*** | DSM 14402$^T$ | seawater | no | 500 | 250 | 250 | | 1000 | 241x | 61 | 16 | 38298 | 2 | 4147593 | 3390388 | 3390388 | 757205 | 40.1 | 106 | 9 | Compl. | 3811 | Compl. | 1 | 757205 | 22.3 | CP011034, CP011035 |
| ***P. tunicata (Ptun)*** | DSM 14096$^T$ | tunicate | yes | 552 | 550 | | 818 | 1920 | 387x | 67 | 43 | 107562 | 2 | 4959443 | 3978968 | 3978968 | 980475 | 39.9 | 107 | 10 | Compl. | 4425 | Compl. | 1 | 980475 | 24.6 | CP011032, CP011033 |
| *P. undina (Pund)* | DSM 6065$^T$ | seawater | no | 520 | 250 | 250 | | 1020 | 253x | 65 | 19 | 40118 | 4 | 4027610 | 2487699 | 2487699 | 637750 | 40 | 98 | 6 | draft | 3692 | Compl. | 1 | 774670 | 23.8 | AHCF00000000 |
| *P. piscicida (Ppis)* | JCM 20779$^T$ | red tide waters | yes | 540 | 250 | 250 | 751 | 1791 | 326x | 65 | 17 | 39523 | 2 | 5488179 | 4253375 | 4253375 | 1234804 | 43.3 | 189 | 9 | draft | 5021 | Compl. | 1 | 1234804 | 29.0 | CP011924, CP011925 |
| *P. aliena (Pali)* | DSM 16473$^T$ | seawater | melanin | 554 | 555 | | | 1109 | 247x | 67 | 11 | 24005 | 29 | 4493055 | 1279857 | 529276 | 160256 | 39 | 99 | 2 | draft | 4102 | Compl. | 1 | 815208 | 22.2 | AQGU00000000 |
| *P. aurantia (Paur)* | DSM 6057$^T$ | seawater | yes | 553 | 552 | | 760 | 1865 | 333x | 65 | 11 | 21410 | 15 | 5602374 | 2589228 | 1267804 | 338164 | 40.8 | 122 | 4 | draft | 4930 | draft | 1 | 1267804 | 29.2 | AQGV00000000 |
| *P. carrageenovora (Pcar)* | DSM 6820$^T$ | seawater/seaweed | no | 551 | 553 | | | 1104 | 242x | 67 | 4 | 8503 | 25 | 4560230 | 1029681 | 700568 | 206280 | 39.4 | 95 | 1 | draft | 4110 | Compl. | 1 | 820986 | 22.0 | AQGW00000000 |
| *P. citrea (Pcit)* | DSM 8771$^T$ | seawater | yes | 540 | | 250 | | 790 | 145x | 65 | 0 | 0 | 27 | 5434598 | 1164454 | 732536 | 203006 | 41.2 | 100 | 3 | draft | 4788 | draft | 1 | 1164454 | 27.3 | AHBZ00000000 |
| *P. flavipulchra (Pfla)* | DSM 14401$^T$ | seawater | yes | 555 | 553 | | 1793 | 2901 | 533x | 63 | 7 | 16413 | 10 | 5441065 | 1858647 | 948187 | 301586 | 43.2 | 152 | 0 | draft | 4919 | draft | 2 | 1249773 | 29.8 | AQGY00000000 |
| *P. lipolytica (Plip)* | JCM 15903$^T$ | estuary | no | 553 | 553 | | 844 | 1950 | 427x | 61 | 2 | 1902 | 36 | 4570506 | 1143391 | 625411 | 264009 | 41.5 | 109 | 0 | draft | 4098 | Compl. | 1 | 898297 | 24.5 | AQHA00000000 |
| *P. luteoviolacea (Plut)* | DSM 6061$^T$ | seawater | yes | 510 | | 250 | 2264 | 3024 | 499x | 55 | 3 | 7108 | 49 | 6062137 | 1228927 | 384841 | 132371 | 41.8 | 210 | 1 | draft | 5346 | Compl. | 1 | 1228927 | 25.4 | AQHB00000000 |
| *P. mariniglutinosa (Pmag)* | DSM 15203$^T$ | diatom | no | 510 | 250 | 250 | 1846 | 2856 | 568x | 53 | 1 | 419 | 27 | 5031620 | 1414176 | 416717 | 142024 | 40.9 | 109 | 1 | draft | 4448 | Compl. | 1 | 1011959 | 25.2 | AQHC00000000 |
| *P. marina (Pman)* | DSM 17587$^T$ | sediment | no | 520 | 250 | 250 | | 1020 | 243x | 65 | 3 | 4279 | 12 | 4190548 | 1931316 | 728006 | 207552 | 39.7 | 102 | 1 | draft | 3861 | Compl. | 1 | 728006 | 21.0 | AHCB00000000 |
| *P. paragorgicola (Ppar)* | DSM 26439$^T$ | sponge | no | 502 | 503 | | | 1005 | 233x | 69 | 4 | 11138 | 22 | 4322351 | 787813 | 759891 | 300025 | 39.2 | 109 | 1 | draft | 3933 | Compl. | 1 | 697863 | 19.3 | AQHE00000000 |
| *P. peptidolytica (Ppep)* | DSM 14001$^T$ | seawater | yes | 500 | | 250 | 2195 | 2945 | 568x | 61 | 1 | 1732 | 34 | 5183489 | 1309995 | 754289 | 138689 | 42.5 | 98 | 1 | draft | 4711 | draft | 2 | 1047960 | 25.3 | AQHF00000000 |
| *P. phenolica (Pphe)* | JCM 21460$^T$ | seawater | yes | 510 | | 502 | 1707 | 2719 | 560x | 65 | 15 | 30112 | 8 | 4852163 | 1297686 | 1007183 | 359234 | 40.5 | 149 | 4 | draft | 4380 | Compl. | 1 | 1023506 | 26.7 | AQHG00000000 |
| *P. prydzensis (Ppry)* | DSM 14232$^T$ | sea ice | no | 500 | | 250 | 1913 | 2663 | 512x | 63 | 1 | 501 | 21 | 5205557 | 1100710 | 916042 | 193899 | 41.2 | 126 | 6 | draft | 4661 | Compl. | 1 | 1099445 | 26.8 | AQHH00000000 |
| *P. rubra (Prub)* | DSM 6842$^T$ | seawater | yes | 600 | 250 | 250 | 1954 | 3054 | 497x | 65 | 4 | 3417 | 44 | 6143755 | 1388597 | 574146 | 135658 | 47.8 | 240 | 1 | draft | 5352 | Compl. | 1 | 1388597 | 29.2 | AHCD00000000 |
| *P. ulvae (Pulv)* | DSM 15557$^T$ | marine alga | yes | 551 | 554 | | 1786 | 2891 | 609x | 61 | 1 | 412 | 35 | 4745459 | 856917 | 462978 | 138119 | 41.2 | 192 | 1 | draft | 4383 | Compl. | 1 | 856917 | 22.0 | AQHJ00000000 |

*Species with complete genome sequences are indicated in bold. Genomes harboring a plasmid are underlined.

**Other SOAPdenovo parameters: rd_len_cutoff=90; pair_num_cutoff=5; map_len=80; avg_ins=478, 2000, 5000, and 10000 for 500-bp, 2-kb, 5-kb, and 10-kb libraries, respectively; reverse_seq=0 for 500-bp library and 1 for other libraries; asm_flags=3 for 500-bp library and 2 for other libraries.
