# Supplementary material for: Evolutionary Trajectory of the Replication Mode of Bacterial Replicons
Source: mBio. 2021 Jan 26;12(1):e02745-20. doi: 10.1128/mBio.02745-20 (PMC7858055; doi:10.1128/mBio.02745-20)
Supplement: TABLE S3 [file mBio.02745-20-st003.pdf]

Table S3. (a) Genes involved in chromosome replication and their homologues in model organisms *V. cholerae* , *E. coli* , and *B. subtilis* . (b) The replication origin sites (*ori*) and termination sites (*dif*) on the main chromosomes and chromids.

a

Function classification	Gene	Chromosome	Gene function	Locus (coordinate) in *P. tunicata*	Prot. len.	BLASTP hit to *V. cholerae*			BLASTP hit to *E. coli*			BLASTP hit to *B. subtilis*		
				and *P. spongiae*	(aa)	Prot. acc.	Len. (aa)	Identity	Prot. acc.	Len. (aa)	Identity	Prot. acc.	Len. (aa)	Identity
Chromosome replication	*dnaA*	1	chromosomal replication initiator protein DnaA	PTUNa0001(167-1549)	460	gi|15640044|ref|NP_062596.1|	472	349/470 (74%)	gi|388479539|ref|YP_491733.1|	467	350/471 (74%)	gi|16077069|ref|NP_387882.1|	446	190/460 (41%)
				PSPOa0001(170-1549)	459	gi|15640044|ref|NP_062596.1|	472	346/464 (74%)	gi|388479539|ref|YP_491733.1|	467	352/471 (74%)	gi|16077069|ref|NP_387882.1|	446	190/448 (42%)
	dnaB	1	replicative DNA helicase	PTUNa0717(670373-671752)	459	gi|15640398|ref|NP_230025.1|	468	345/455 (75%)	gi|388480001|ref|YP_492195.1|	471	350/454 (77%)	gi|255767872|ref|NP_391924.2|	454	207/447 (46%)
				PSPOa2614(2504848-2506227)	459	gi|15640398|ref|NP_230025.1|	468	344/454 (75%)	gi|388480001|ref|YP_492195.1|	471	351/454 (77%)	gi|255767872|ref|NP_391924.2|	454	205/439 (46%)
	dnaN	1	DNA polymerase III subunit beta	PTUNa0002(1560-2666)	364	gi|15640046|ref|NP_062598.1|	363	135/364 (37%)	gi|388479540|ref|YP_491734.1|	366	197/367 (53%)	gi|16077070|ref|NP_387883.1|	378	95/377 (25%)
				PSPOa0002(1562-2665)	364	gi|15640046|ref|NP_062598.1|	363	146/364 (40%)	gi|388479540|ref|YP_491734.1|	366	198/367 (53%)	gi|16077070|ref|NP_387883.1|	378	96/380 (25%)
	dnaE	1	DNA polymerase III subunit alpha	PTUNa2966(2728888-2732379)	1163	gi|15642243|ref|NP_231876.1|	1164	800/1164 (68%)	gi|388476302|ref|YP_488486.1|	1160	786/1165 (67%)	gi|16079975|ref|NP_390801.1|	1115	427/1158 (36%)
				PSPOa2108(2002561-2006052)	1163	gi|15642243|ref|NP_231876.1|	1164	809/1164 (69%)	gi|388476302|ref|YP_488486.1|	1160	805/1165 (69%)	gi|16079975|ref|NP_390801.1|	1115	427/1156 (36%)
	dnaQ	1	DNA polymerase III subunit epsilon	PTUNa2786(2555182-2555940)	252	gi|15642231|ref|NP_231864.1|	247	137/239 (57%)	gi|388476328|ref|YP_488512.1|	243	137/238 (57%)	-	-	-
				PSPOa1196(1121114-1121830)	238	gi|15642231|ref|NP_231864.1|	247	136/237 (57%)	gi|388476328|ref|YP_488512.1|	243	143/235 (60%)	-	-	-
	dnaX	1	DNA polymerase III subunit gamma/tau	PTUNa2869(2629248-2631710)	820	gi|15641067|ref|NP_230699.1|	692	214/369 (57%)	gi|388476575|ref|YP_488761.1|	643	214/365 (58%)	gi|255767015|ref|NP_387900.2|	563	130/277 (46%)
				PSPOa2040(1936622-1938907)	761	gi|15641067|ref|NP_230699.1|	692	210/361 (58%)	gi|388476575|ref|YP_488761.1|	643	219/363 (60%)	gi|255767015|ref|NP_387900.2|	563	121/226 (53%)
	holA	1	DNA polymerase III subunit delta	PTUNa1467(1342707-1343747)	346	gi|15640969|ref|NP_230600.1|	342	94/307 (30%)	gi|388476743|ref|YP_488931.1|	343	96/332 (28%)	gi|16079610|ref|NP_390434.1|	347	63/314 (20%)
				PSPOa1265(1182554-1183609)	351	gi|15640969|ref|NP_230600.1|	342	104/334 (31%)	gi|388476743|ref|YP_488931.1|	343	105/314 (33%)	gi|16079610|ref|NP_390434.1|	347	66/318 (20%)
	holB	1	DNA polymerase III subunit delta'	PTUNa2495(2309851-2310759)	302	gi|15642017|ref|NP_231649.1|	324	51/174 (29%)	gi|388477179|ref|YP_489367.1|	334	60/175 (34%)	gi|16077099|ref|NP_387912.1|	329	35/167 (20%)
				PPSPOa1794(1692766-1693656)	296	gi|15642017|ref|NP_231649.1|	324	50/131 (38%)	gi|388477179|ref|YP_489367.1|	334	51/126 (40%)	gi|16077099|ref|NP_387912.1|	329	45/125 (36%)
	holC	1	DNA polymerase III subunit chi	PTUNa0922(828560-829000)	146	gi|15642498|ref|NP_232131.1|	149	50/114 (43%)	gi|388480202|ref|YP_492397.1|	147	64/142 (45%)	-	-	-
				PSPOa2267(2174640-2175080)	146	gi|15642498|ref|NP_232131.1|	149	50/113 (44%)	gi|388480202|ref|YP_492397.1|	147	60/142 (42%)	-	-	-
	recF	1	DNA replication and repair protein RecF	PTUNa0003(2669-3763)	364	gi|15640046|ref|NP_062598.1|	363	135/364 (37%)	gi|388479541|ref|YP_491735.1|	357	141/363 (38%)	gi|16077072|ref|NP_387885.1|	370	96/380 (25%)
				PSPOa0003(2688-3782)	364	gi|15640046|ref|NP_062598.1|	363	146/364 (40%)	gi|388479541|ref|YP_491735.1|	357	156/361 (43%)	gi|16077072|ref|NP_387885.1|	370	106/376 (28%)
	gyrB	1	DNA gyrase, subunit B	PTUNa0004(3890-6193)	767	gi|471237023|ref|NP_229675.2|	805	520/768 (67%)	gi|388479542|ref|YP_491736.1|	804	522/768 (67%)	gi|16077074|ref|NP_387887.1|	638	279/515 (54%)
				PSPOa0004(3795-6212)	805	gi|471237023|ref|NP_229675.2|	805	573/806 (71%)	gi|388479542|ref|YP_491736.1|	804	570/806 (70%)	gi|16077074|ref|NP_387887.1|	638	303/560 (54%)
	dnaG	1	DNA primase	PTUNa3488(3207976-3209727)	583	gi|15642769|ref|NP_230169.1|	587	296/589 (50%)	gi|388479066|ref|YP_491258.1|	581	297/584 (50%)	gi|255767574|ref|NP_390400.2|	603	134/372 (36%)
				PSPOa0542(526166-527911)	581	gi|15642769|ref|NP_230169.1|	587	296/589 (50%)	gi|388479066|ref|YP_491258.1|	581	282/580 (48%)	gi|255767574|ref|NP_390400.2|	603	155/463 (33%)
	ssb	1	single-strand DNA-binding protein (SSB)	PTUNa0402(384573-385289)	238	gi|15640424|ref|NP_230051.1|	177	79/117 (67%)	gi|388480008|ref|YP_492202.1|	178	84/115 (73%)	gi|16081142|ref|NP_391970.1|	172	37/97 (38%)
				PSPOa3033(2912662-2913336)	224	gi|15640424|ref|NP_230051.1|	177	83/115 (72%)	gi|388480008|ref|YP_492202.1|	178	85/115 (73%)	gi|16081142|ref|NP_391970.1|	172	40/100 (40%)
Chromosome partitioning system	*parA* 1	1	chromosome partitioning protein, ATPases	PTUNa4277(3969013-3969798)	261	gi|15642766|ref|NP_232399.1|	257	203/254 (79%)	-	-	-	gi|16081149|ref|NP_391977.1|	253	131/250 (52%)
				PSPOa3274(3137940-3138725)	261	gi|15642766|ref|NP_232399.1|	257	202/254 (79%)	-	-	-	gi|16081149|ref|NP_391977.1|	253	125/249 (50%)
	parB 1	1	chromosome partitioning protein, ParB family	PTUNa4275(3968068-3968991)	307	gi|15642765|ref|NP_232398.1|	293	154/299 (51%)	-	-	-	gi|16081148|ref|NP_391976.1|	282	102/297 (34%)
				PSPOa3273(3137015-3137923)	302	gi|15642765|ref|NP_232398.1|	293	151/297 (50%)	-	-	-	gi|16081148|ref|NP_391976.1|	282	103/299 (34%)
Chromosome segregation system	*smc*	1	structural maintenance of chromosomes (SMC)	PTUNa1539(1402684-1406109)	1141	-	-	-	-	-	-	gi|255767364|ref|NP_389476.2|	1186	100/205 (48%)
				PSPOa1983(1879216-1882638)	1140	-	-	-	-	-	-	gi|255767364|ref|NP_389476.2|	1186	93/193 (48%)
	scpA	1	segregation and condensation protein A	PTUNa1701(1555841-1556665)	274	-	-	-	-	-	-	gi|16079379|ref|NP_390203.1|	251	93/245 (37%)
				PSPOa1353(1271939-1272733)	264	-	-	-	-	-	-	gi|16079379|ref|NP_390203.1|	251	87/245 (35%)
	scpB	1	segregation and condensation protein B	PTUNa1702(1556766-1557356)	196	-	-	-	-	-	-	gi|16079378|ref|NP_390202.1|	197	60/171 (35%)
				PSPOa1354(1272751-1273329)	192	-	-	-	-	-	-	gi|16079378|ref|NP_390202.1|	197	43/123 (34%)
Chromosome dimer resolution system	*ftsK*	1	DNA translocase, CDR system	PTUNa1954(1816000-1818516)	838	gi|15641905|ref|NP_231537.1|	960	337/479 (70%)	gi|388476974|ref|YP_489162.1|	1329	325/479 (67%)	gi|255767684|ref|NP_390859.2|	952	228/467 (48%)
				PSPOa1556(1454845-1457451)	868	gi|15641905|ref|NP_231537.1|	960	346/493 (70%)	gi|388476974|ref|YP_489162.1|	1329	334/495 (67%)	gi|255767684|ref|NP_390859.2|	952	219/462 (47%)
	xerC	1	site-specific tyrosine recombinase, CDR system	PTUNa0077(84852-85769)	305	gi|15640159|ref|NP_229786.1|	311	149/303 (49%)	gi|388479438|ref|YP_491630.1|	298	143/293 (48%)	gi|16078677|ref|NP_389496.1|	304	113/300 (37%)
				PSPOa0046(49088-50005)	305	gi|15640159|ref|NP_229786.1|	311	143/304 (47%)	gi|388479438|ref|YP_491630.1|	298	142/292 (48%)	gi|16078677|ref|NP_389496.1|	304	109/298 (36%)
	xerD	1	site-specific tyrosine recombinase, CDR system	PTUNa1087(1002754-1003659)	301	gi|15642416|ref|NP_232049.1|	302	172/294 (58%)	gi|388478903|ref|YP_491095.1|	298	180/291 (61%)	gi|255767509|ref|NP_390232.2|	296	131/292 (44%)
				PSPOa0808(770548-771453)	301	gi|15642416|ref|NP_232049.1|	302	172/291 (59%)	gi|388478903|ref|YP_491095.1|	298	174/291 (59%)	gi|255767509|ref|NP_390232.2|	296	130/292 (44%)
Plasmid replication	*repA*	2 (Chromid)	plasmid replication initiator protein (repA-like)	PTUNb0959(978599-979813)	404	-	-	-	-	-	-	-	-	-
				PSPOb1826(1596324-1597541)	405	-	-	-	-	-	-	-	-	-
	ywqA	2(Chromid)	DNA helicase	PTUNb0004(2597-5779)	1060	-	-	-	-	-	-	gi|16080681|ref|NP_391509.1|	922	194/486 (39%)
				PSPOb0004(2594-5725)	1043	-	-	-	-	-	-	gi|16080681|ref|NP_391509.1|	922	194/475 (40%)
Plasmid partitioning system	*parA* 2	2(Chromid)	plasmid partitioning protein, ATPases	PTUNb0001(161-1396)	411	gi|15601863|ref|NP_233494.1|	407	116/393 (29%)	-	-	-	-	-	-
				PSPOb0001(161-1396)	411	gi|15601863|ref|NP_233494.1|	407	107/395 (27%)	-	-	-	-	-	-
	parB 2	2(Chromid)	plasmid partitioning protein, ParB family	PTUNb0003(1410-2381)	323	gi|15601862|ref|NP_233493.1|	323	69/248 (27%)	-	-	-	-	-	-

				PSPOb0002(1410-2378)	322	gi|15601862|ref|NP_233493.1|	323	63/204 (30%)	-	-	-	-	-	-
DNA repair	*rmuC*	2(Chromid)	DNA recombination protein RmuC	PTUNb0010(9496-11058)	520	gi|15640114|ref|NP_229741.1|	513	143/377 (37%)	gi|388479418|ref|YP_491610.1|	475	144/394 (36%)	-	-	-
				PSPOb0008(7290-8648)	452	gi|15640114|ref|NP_229741.1|	513	152/382 (39%)	gi|388479418|ref|YP_491610.1|	475	135/356 (37%)	-	-	-
Cell division	*minC*	2(Chromid)	septum site-determining protein MinC	PTUNb0847(878407-879108)	233	gi|15641961|ref|NP_231593.1|	220	103/226 (45%)	gi|388477255|ref|YP_489443.1|	231	96/236 (40%)	gi|16079852|ref|NP_390678.1|	226	24/67 (35%)
				PSPOb0844(783586-784284)	232	gi|15641961|ref|NP_231593.1|	220	103/228 (45%)	gi|388477255|ref|YP_489443.1|	231	101/237 (42%)	gi|16079852|ref|NP_390678.1|	226	28/70 (40%)
	minD	2(Chromid)	septum site-determining protein MinD	PTUNb0846(877592-878401)	269	gi|15641962|ref|NP_231594.1|	276	200/250 (80%)	gi|388477254|ref|YP_489442.1|	270	188/250 (75%)	gi|16079851|ref|NP_390677.1|	268	103/248 (41%)
				PSPOb0843(782764-783573)	269	gi|15641962|ref|NP_231594.1|	276	193/250 (77%)	gi|388477254|ref|YP_489442.1|	270	190/250 (76%)	gi|16079851|ref|NP_390677.1|	268	102/247 (41%)
	minE	2(Chromid)	septum formation topological specificity factor	PTUNb0845(877327-877590)	87	gi|15641963|ref|NP_231595.1|	87	55/87 (63%)	gi|388477253|ref|YP_489441.1|	88	52/83 (62%)	-	-	-
				PSPOb0842(782505-782762)	85	gi|15641963|ref|NP_231595.1|	87	54/85 (63%)	gi|388477253|ref|YP_489441.1|	88	53/83 (63%)	-	-	-
Replication termination	*tus*	2(Chromid)	DNA replication terminus site-binding protein	PTUNb0958(977664-978539)	291	-	-	-	gi|388477685|ref|YP_489873.1|	309	70/261 (26%)	-	-	-
				PSPOb1825(1595454-1596317)	287	-	-	-	gi|388477685|ref|YP_489873.1|	309	91/277 (32%)	-	-	-

b

Species/Strains	Replicon size	*ori* site								*dif* site		Relative position%*
		Start	End	Len.	AT%	Num. of DnaA boxes	Num. of Dam methylase sites (GATC)	Gene upwards	Gene downwards	Start	End	
Main chromosome (Chr1)												
V. cholerae	2961149	2961047	371	474	63.7	5	14	gidA	mioC	1564104	1564131	47.2
P. agarivorans DSM14585^{T}	3701940	3695595	3696020	426	68.1	4	13	gidA	mioC	1890775	1890802	48.9
P. espejiana DSM9414^{T}	3720756	3714077	3714503	427	67.4	4	14	gidA	mioC	1863910	1863937	49.9
P. arctica DSM18437^{T}	3840834	3834376	3834803	428	69.4	4	14	gidA	mioC	1973222	1973249	48.6
P. translucida DSM14402^{T}	3390388	3383995	3384420	426	67.8	4	13	gidA	mioC	1733390	1733417	48.9
P. haloplanktis TAC125	3214944	3208603	3209028	426	67.8	4	13	gidA	mioC	1580506	1580533	49.2
Pseudoalteromonas sp. SM9913	3332787	3323019	3323446	428	68.9	3	15	gidA	mioC	1653741	1653768	50.1
P. nigrifaciens DSM8810^{T}	3521200	3514821	3515246	426	68.3	4	13	gidA	mioC	1767602	1767629	49.8
P. issachenkonii DSM15925^{T}	3403660	3393894	3394321	428	69.2	4	15	gidA	mioC	1664828	1664855	48.9
P. tetraodonis DSM9166^{T}	3399143	3389374	3389801	428	69.4	4	15	gidA	mioC	1719261	1719288	49.4
P. tunicata DSM14096^{T}	3978968	3972339	3972766	428	66.8	4	13	gidA	mioC	1940591	1940618	48.8
P. spongiae JCM12884^{T}	3154175	3141283	3141710	428	70.3	4	12	gidA	mioC	1540643	1540670	48.8
Pseudoalteromonas sp. SAO4-4	3282406	3269512	3269939	428	69.9	2	13	gidA	mioC	1572722	1572695	51.7
P. piratica OCN003^{T}	3197498	713574	713998	425	70.8	4	14	gidA	mioC	2325112	2325139	49.6
Chromid (Chr2)												
V. cholerae	1072315	247	1133	887	61.9	1	20	parA	rctB	507983	508010	47.3
P. agarivorans DSM14585^{T}	843022	842409	159	773	72.1	3	12	repA	parA	827850	827877	1.8
P. espejiana DSM9414^{T}	779695	779082	160	774	71.2	2	12	repA	parA	763820	763847	2.0
P. carrageenovora DSM6820^{T}	820986	820308	160	839	71	2	12	repA	parA	805701	805728	1.9
P. marina DSM17587^{T}	728006	727393	161	775	72.5	2	12	repA	parA	713172	713199	2.0
P. aliena DSM16473^{T}	815208	814595	161	775	71.6	2	12	repA	parA	799620	799647	1.9
P. arctica DSM18437^{T}	783876	783198	160	839	69.5	2	12	repA	parA	768184	768211	2.0
P. paragorgicola DSM26439^{T}	697863	697185	160	839	69.6	2	12	repA	parA	682560	682587	2.2
P. translucida DSM14402^{T}	757205	756608	159	757	72.5	1	12	repA	parA	741645	741672	2.1
P. haloplanktis TAC125	635328	634716	159	772	71.9	1	12	repA	parA	619752	619779	2.5
Pseudoalteromonas sp. SM9913	704884	704272	160	773	72.2	1	12	repA	parA	690010	690037	2.0
P. nigrifaciens DSM8810^{T}	679846	679249	159	757	72.1	1	12	repA	parA	664270	664297	2.3
P. undina DSM6065^{T}	774670	774059	159	771	70.8	1	12	repA	parA	759783	759810	1.9
P. issachenkonii DSM15925^{T}	728958	728346	160	773	72.3	1	12	repA	parA	714081	714108	2.0
P. tetraodonis DSM9166^{T}	729257	728645	160	773	72.2	1	12	repA	parA	714383	714410	2.0

P. prydzensis DSM14232^T	1099445	1098833	160	773	71.8	4	14	repA	parA	1083960	1083987	1.4
P. mariniglutinosa DSM15203^T	1011959	1011347	160	773	72.8	4	14	repA	parA	996295	996322	1.5
P. lipolytica JCM15903^T	898297	897664	160	794	71.3	3	13	repA	parA	883608	883635	1.6
P. phenolica JCM21460^T	1023506	1022993	160	674	74.8	2	11	repA	parA	1008444	1008471	1.5
P. luteoviolacea DSM6061^T	1228927	1228380	160	708	73.7	3	13	repA	parA	1224187	1224214	0.3
P. rubra DSM6842^T	1388597	1388057	160	701	68.9	3	16	repA	parA	1360943	1360970	2.0
P. piscicida JCM20779^T	1234804	1234263	160	702	70.9	3	14	repA	parA	1228213	1228240	0.5
P. ulvae DSM15557^T	856917	856249	160	829	73.9	1	15	repA	parA	840939	840966	1.9
P. tunicata DSM14096^T	980475	979814	160	822	73.8	2	13	repA	parA	964729	964756	1.6
P. spongiae JCM12884^T	1598238	1597542	160	857	70.1	2	15	repA	parA	819264	819291	48.7
Pseudoalteromonas sp. SAO4-4	1667761	1667065	160	857	70	2	15	repA	parA	828218	828245	49.7
P. piratica OCN003^T	1618489	78000	78855	856	70.9	2	14	repA	parA	862562	862589	48.5

*Relative position% is calculated as distance-between-ori2-and-dif2 / replicon-size * 100%. A value close to 0 implies unidirectional replication. A value close to 50% implies bidirectional replication.
