# Supplementary material for: Evolutionary Trajectory of the Replication Mode of Bacterial Replicons
Source: mBio. 2021 Jan 26;12(1):e02745-20. doi: 10.1128/mBio.02745-20 (PMC7858055; doi:10.1128/mBio.02745-20)
Supplement: FIG S2 [file mBio.02745-20-sf002.pdf]

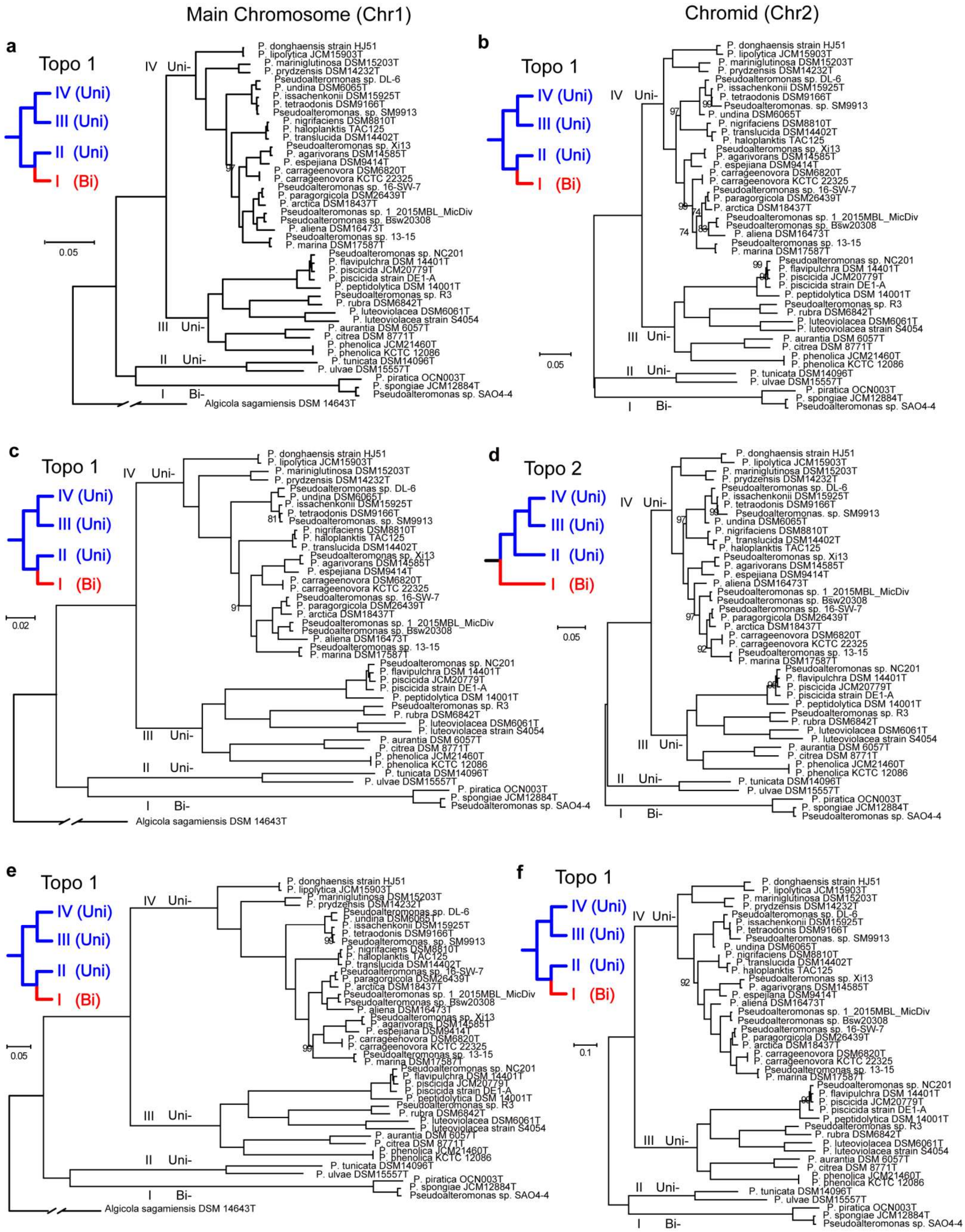
Main Chromosome (Chr1)
Chromid (Chr2)
a
Topo 1
IV (Uni)
III (Uni)
II (Uni)
I (Bi)
0.05
IV Uni-
P. donghaensis strain HJ51
P. lipolytica JCM15903T
P. mariniglutinosa DSM15203T
P. prydzensis DSM14232T
Pseudoalteromonas sp. DL-6
P. undina DSM6065T
P. issachenkonii DSM15925T
P. tetraodonis DSM9166T
Pseudoalteromonas. sp. SM9913
P. nigrifaciens DSM8810T
P. haloplanktis TAC125
P. translucida DSM14402T
Pseudoalteromonas sp. Xi13
P. agarivorans DSM14585T
P. espejiana DSM9414T
97
P. carrageenovora DSM6820T
P. carrageenovora KCTC 22325
Pseudoalteromonas sp. 16-SW-7
P. paragorgicola DSM26439T
P. arctica DSM18437T
Pseudoalteromonas sp. 1_2015MBL_MicDiv
Pseudoalteromonas sp. Bsw20308
P. aliena DSM16473T
Pseudoalteromonas sp. 13-15
P. marina DSM17587T
Pseudoalteromonas sp. NC201
P. flavipulchra DSM 14401T
P. piscicida JCM20779T
P. piscicida strain DE1-A
P. peptidolytica DSM 14001T
Pseudoalteromonas sp. R3
P. rubra DSM6842T
P. luteoviolacea DSM6061T
P. luteoviolacea strain S4054
III Uni-
P. aurantia DSM 6057T
P. citrea DSM 8771T
P. phenolica JCM21460T
P. phenolica KCTC 12086
II Uni-
P. tunicata DSM14096T
P. ulvae DSM15557T
P. piratica OCN003T
P. spongiae JCM12884T
Pseudoalteromonas sp. SAO4-4
I Bi-
Algicola sagamiensis DSM 14643T
b
Topo 1
IV (Uni)
III (Uni)
II (Uni)
I (Bi)
0.05
IV Uni-
P. donghaensis strain HJ51
P. lipolytica JCM15903T
P. mariniglutinosa DSM15203T
P. prydzensis DSM14232T
Pseudoalteromonas sp. DL-6
P. issachenkonii DSM15925T
P. tetraodonis DSM9166T
99
Pseudoalteromonas. sp. SM9913
97
P. undina DSM6065T
P. nigrifaciens DSM8810T
P. translucida DSM14402T
P. haloplanktis TAC125
Pseudoalteromonas sp. Xi13
P. agarivorans DSM14585T
P. espejiana DSM9414T
P. carrageenovora DSM6820T
P. carrageenovora KCTC 22325
Pseudoalteromonas sp. 16-SW-7
P. paragorgicola DSM26439T
99
74
P. arctica DSM18437T
Pseudoalteromonas sp. 1_2015MBL_MicDiv
Pseudoalteromonas sp. Bsw20308
74
83
P. aliena DSM16473T
Pseudoalteromonas sp. 13-15
P. marina DSM17587T
Pseudoalteromonas sp. NC201
99
P. flavipulchra DSM 14401T
99
P. piscicida JCM20779T
P. piscicida strain DE1-A
P. peptidolytica DSM 14001T
Pseudoalteromonas sp. R3
P. rubra DSM6842T
P. luteoviolacea DSM6061T
P. luteoviolacea strain S4054
III Uni-
P. aurantia DSM 6057T
P. citrea DSM 8771T
P. phenolica JCM21460T
P. phenolica KCTC 12086
II Uni-
P. tunicata DSM14096T
P. ulvae DSM15557T
P. piratica OCN003T
P. spongiae JCM12884T
Pseudoalteromonas sp. SAO4-4
I Bi-
c
Topo 1
IV (Uni)
III (Uni)
II (Uni)
I (Bi)
0.02
IV Uni-
P. donghaensis strain HJ51
P. lipolytica JCM15903T
P. mariniglutinosa DSM15203T
P. prydzensis DSM14232T
Pseudoalteromonas sp. DL-6
P. undina DSM6065T
P. issachenkonii DSM15925T
P. tetraodonis DSM9166T
81
Pseudoalteromonas. sp. SM9913
P. nigrifaciens DSM8810T
P. haloplanktis TAC125
P. translucida DSM14402T
Pseudoalteromonas sp. Xi13
P. agarivorans DSM14585T
P. espejiana DSM9414T
P. carrageenovora DSM6820T
P. carrageenovora KCTC 22325
Pseudoalteromonas sp. 16-SW-7
P. paragorgicola DSM26439T
91
P. arctica DSM18437T
Pseudoalteromonas sp. 1_2015MBL_MicDiv
Pseudoalteromonas sp. Bsw20308
P. aliena DSM16473T
Pseudoalteromonas sp. 13-15
P. marina DSM17587T
Pseudoalteromonas sp. NC201
P. flavipulchra DSM 14401T
P. piscicida JCM20779T
P. piscicida strain DE1-A
P. peptidolytica DSM 14001T
Pseudoalteromonas sp. R3
P. rubra DSM6842T
P. luteoviolacea DSM6061T
P. luteoviolacea strain S4054
III Uni-
P. aurantia DSM 6057T
P. citrea DSM 8771T
P. phenolica JCM21460T
P. phenolica KCTC 12086
II Uni-
P. tunicata DSM14096T
P. ulvae DSM15557T
P. piratica OCN003T
P. spongiae JCM12884T
Pseudoalteromonas sp. SAO4-4
I Bi-
Algicola sagamiensis DSM 14643T
d
Topo 2
IV (Uni)
III (Uni)
II (Uni)
I (Bi)
0.05
IV Uni-
P. donghaensis strain HJ51
P. lipolytica JCM15903T
P. mariniglutinosa DSM15203T
P. prydzensis DSM14232T
Pseudoalteromonas sp. DL-6
P. issachenkonii DSM15925T
P. tetraodonis DSM9166T
99
Pseudoalteromonas. sp. SM9913
97
P. undina DSM6065T
P. nigrifaciens DSM8810T
P. translucida DSM14402T
P. haloplanktis TAC125
Pseudoalteromonas sp. Xi13
P. agarivorans DSM14585T
P. espejiana DSM9414T
P. aliena DSM16473T
Pseudoalteromonas sp. 1_2015MBL_MicDiv
Pseudoalteromonas sp. Bsw20308
Pseudoalteromonas sp. 16-SW-7
97
P. paragorgicola DSM26439T
P. arctica DSM18437T
P. carrageenovora DSM6820T
92
P. carrageenovora KCTC 22325
Pseudoalteromonas sp. 13-15
P. marina DSM17587T
Pseudoalteromonas sp. NC201
P. flavipulchra DSM 14401T
99
P. piscicida JCM20779T
P. piscicida strain DE1-A
P. peptidolytica DSM 14001T
Pseudoalteromonas sp. R3
P. rubra DSM6842T
P. luteoviolacea DSM6061T
P. luteoviolacea strain S4054
III Uni-
P. aurantia DSM 6057T
P. citrea DSM 8771T
P. phenolica JCM21460T
P. phenolica KCTC 12086
II Uni-
P. tunicata DSM14096T
P. ulvae DSM15557T
P. piratica OCN003T
P. spongiae JCM12884T
Pseudoalteromonas sp. SAO4-4
I Bi-
e
Topo 1
IV (Uni)
III (Uni)
II (Uni)
I (Bi)
0.05
IV Uni-
P. donghaensis strain HJ51
P. lipolytica JCM15903T
P. mariniglutinosa DSM15203T
P. prydzensis DSM14232T
Pseudoalteromonas sp. DL-6
P. undina DSM6065T
P. issachenkonii DSM15925T
P. tetraodonis DSM9166T
99
Pseudoalteromonas. sp. SM9913
P. nigrifaciens DSM8810T
P. haloplanktis TAC125
P. translucida DSM14402T
Pseudoalteromonas sp. 16-SW-7
P. paragorgicola DSM26439T
P. arctica DSM18437T
Pseudoalteromonas sp. 1_2015MBL_MicDiv
Pseudoalteromonas sp. Bsw20308
P. aliena DSM16473T
Pseudoalteromonas sp. Xi13
P. agarivorans DSM14585T
P. espejiana DSM9414T
P. carrageenovora DSM6820T
99
P. carrageenovora KCTC 22325
Pseudoalteromonas sp. 13-15
P. marina DSM17587T
Pseudoalteromonas sp. NC201
P. flavipulchra DSM 14401T
P. piscicida JCM20779T
P. piscicida strain DE1-A
P. peptidolytica DSM 14001T
Pseudoalteromonas sp. R3
P. rubra DSM6842T
P. luteoviolacea DSM6061T
P. luteoviolacea strain S4054
III Uni-
P. aurantia DSM 6057T
P. citrea DSM 8771T
P. phenolica JCM21460T
P. phenolica KCTC 12086
II Uni-
P. tunicata DSM14096T
P. ulvae DSM15557T
P. piratica OCN003T
P. spongiae JCM12884T
Pseudoalteromonas sp. SAO4-4
I Bi-
Algicola sagamiensis DSM 14643T
f
Topo 1
IV (Uni)
III (Uni)
II (Uni)
I (Bi)
0.1
IV Uni-
P. donghaensis strain HJ51
P. lipolytica JCM15903T
P. mariniglutinosa DSM15203T
P. prydzensis DSM14232T
Pseudoalteromonas sp. DL-6
P. issachenkonii DSM15925T
P. tetraodonis DSM9166T
Pseudoalteromonas. sp. SM9913
P. undina DSM6065T
P. nigrifaciens DSM8810T
P. translucida DSM14402T
P. haloplanktis TAC125
92
Pseudoalteromonas sp. Xi13
P. agarivorans DSM14585T
P. espejiana DSM9414T
P. aliena DSM16473T
Pseudoalteromonas sp. 1_2015MBL_MicDiv
Pseudoalteromonas sp. Bsw20308
Pseudoalteromonas sp. 16-SW-7
P. paragorgicola DSM26439T
P. arctica DSM18437T
P. carrageenovora DSM6820T
P. carrageenovora KCTC 22325
Pseudoalteromonas sp. 13-15
P. marina DSM17587T
Pseudoalteromonas sp. NC201
P. flavipulchra DSM 14401T
99
P. piscicida JCM20779T
P. piscicida strain DE1-A
P. peptidolytica DSM 14001T
Pseudoalteromonas sp. R3
P. rubra DSM6842T
P. luteoviolacea DSM6061T
P. luteoviolacea strain S4054
III Uni-
P. aurantia DSM 6057T
P. citrea DSM 8771T
P. phenolica JCM21460T
P. phenolica KCTC 12086
II Uni-
P. tunicata DSM14096T
P. ulvae DSM15557T
P. piratica OCN003T
P. spongiae JCM12884T
Pseudoalteromonas sp. SAO4-4
I Bi-

g

Dataset	Topo	logL	deltaL	bpRELL	pKH	pSH	pWKH	pWSH	cELW	pAU
**Trimming + compositional homogeneous genes**										
	1	-2235995.419	0	1	1	1	1	1	1	1
	2	-2236156.231	160.81	0	0	0	0	0	1.90E-40	7.20E-35
**Trimming**										
	1	-2527573.545	0	1	1	1	1	1	1	1
	2	-2527791.019	217.47	0	0	0	0	0	3.63E-44	4.22E-39
**No Trimming**										
	1	-4279436.473	0	1	1	1	1	1	1	1
	2	-4279601.12	164.65	0	0	0	0	0	3.56E-25	2.77E-05

Supplementary Figure S2. Maximum likelihood trees based on the concatenated amino acid sequences of single-copy genes on the main chromosome (a, c, and e) and chromid (b, d, and f) based on different alignment trimming strategies and the removal of compositionally heterogeneous genes. (a and b). Trimming + compositional homogenous genes. (c and d). Trimming. (e and f). No trimming. The IQ-Tree ultrafast bootstrap percentages are shown on the branches, with 100% not shown for clarity. The main chromosome trees were rooted based on the outgroup *Algicola sagamiensis*. Note that the tree topology (Topo 1) for the main chromosome is robust to different sequence processing methods. The chromid trees, shown by placing the root at the midpoint, were essentially unrooted due to the lack of outgroup. Despite of this, the topology of chromid trees is generally similar to that of main chromosome trees (Topo 1, b and f; Topo 2, d). The bar represents 0.05 substitutions per site. (**g) Topology tests on two different topologies for the chromid trees.** deltaL: logL difference from the maximal logl in the set. bp-RELL: bootstrap proportion using RELL method. p-KH: p-value of one sided Kishino-Hasegawa test. p-SH: p-value of Shimodaira-Hasegawa test. p-WKH: p-value of weighted KH test. p-WSH: p-value of weighted SH test. c-ELW: Expected Likelihood Weight. p-AU: p-value of approximately unbiased (AU) test.
