# Supplementary material for: Evolutionary Trajectory of the Replication Mode of Bacterial Replicons
Source: mBio. 2021 Jan 26;12(1):e02745-20. doi: 10.1128/mBio.02745-20 (PMC7858055; doi:10.1128/mBio.02745-20)
Supplement: FIG S3 [file mBio.02745-20-sf003.pdf]

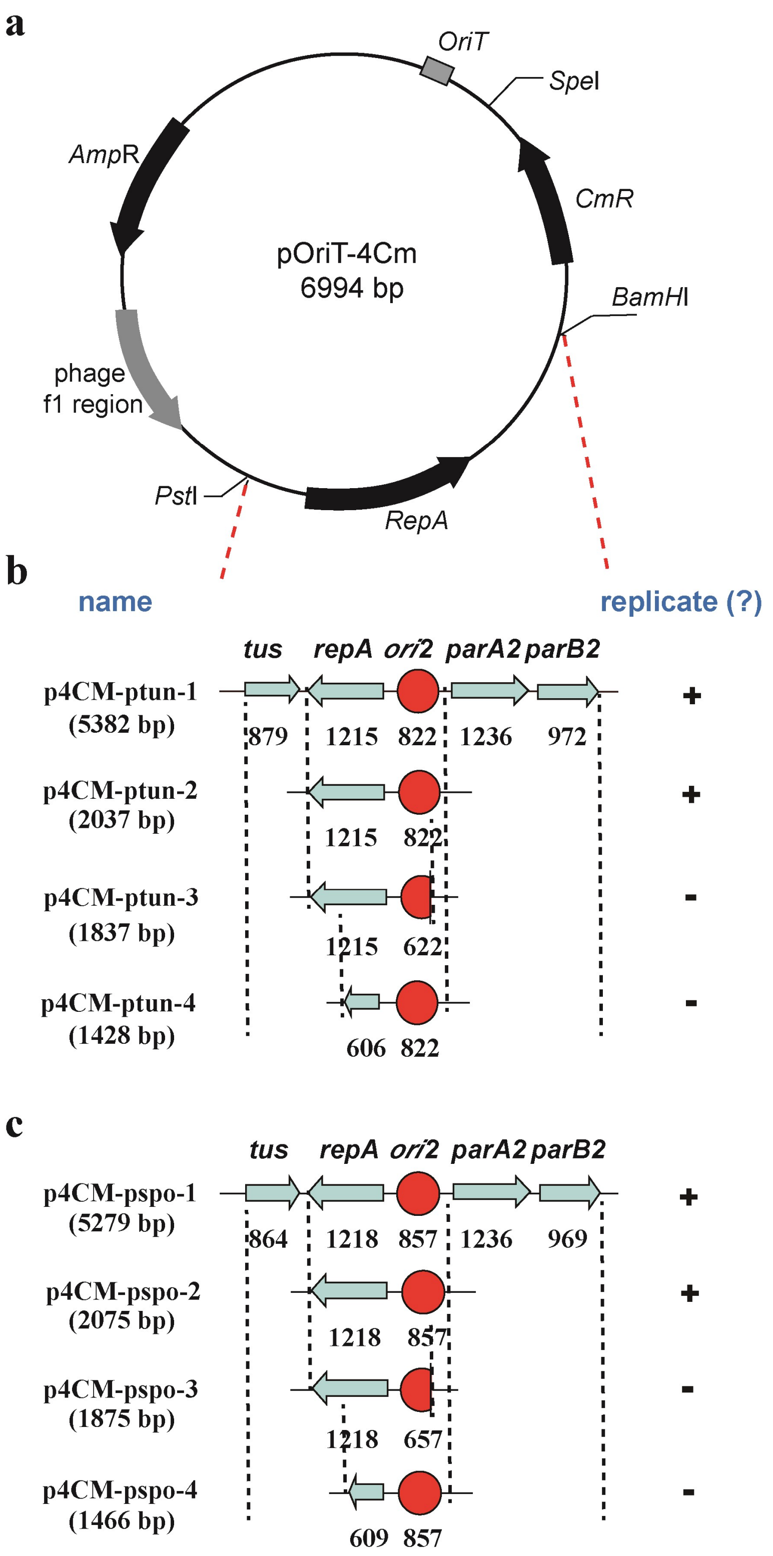

d

Strains/plasmids	Description
Escherichia coli DH5α	gene cloning, 37°C
Escherichia coli WM3064	RP4 (tra) in chromosome, DAP-, 37°C

P. spongiae JCM 12884^T	Host for p4CM-pspo-1, p4CM-pspo-2, p4CM-pspo-3 and p4CM-pspo-4
P. tunicata DSM14096^T	Host for p4CM-ptun-1, p4CM-ptun-2, p4CM-ptun-3 and p4CM-ptun-4
pOriT-4CM	Shuttle vector that constructed previously; template for p4CM
p4CM	A 4898 bp vector constructed by deleting the RepA fragment from pOriT-4CM
p4CM-pspo-1	p4CM + the 5279-bp PCR product (p4CMPs- p4CMBs)<br>Primer p4CMPs: CCCATATGGTCGACCTGCAGGAAACAATTTAACTGATTGG<br>Primer p4CMBs: ACGGGGTGGTGCGTGGATCCCTAGTTAAAGTTAGCAAGTA
p4CM-pspo-2	p4CM + the 2075-bp PCR product (p4CMPros- p4CMBros)<br>Primer p4CMPros: CCCATATGGTCGACCTGCAGTTAATCTTCAACGATTTGTA<br>Primer p4CMBros: ACGGGGTGGTGCGTGGATCCAGCTAAACCTTAACACTTAC
p4CM-pspo-3	p4CM + the 1466-bp PCR product (p4CMPros- p4CMBro200s)<br>Primer p4CMPros: CCCATATGGTCGACCTGCAGTTAATCTTCAACGATTTGTA<br>Primer p4CMBro200s: ACGGGGTGGTGCGTGGATCCACTGTTTGTAAAACATCATT
p4CM-pspo-4	p4CM + the 1875-bp PCR product (p4CMP1/2ros- p4CMBros)<br>Primer p4CMP1/2ros: CCCATATGGTCGACCTGCAGGGTATCTTTCGCTGAATATT<br>Primer p4CMBros: ACGGGGTGGTGCGTGGATCCAGCTAAACCTTAACACTTAC
p4CM-ptun-1	p4CM + the 5382-bp PCR product (p4CMPt- p4CMBt)<br>Primer p4CMPt: CCCATATGGTCGACCTGCAGTTAAATTCTCCTTTAAAGAA<br>Primer p4CMBt: ACGGGGTGGTGCGTGGATCCTTAATTAAAATTCGCAAGTA
p4CM-ptun-2	p4CM + the 2037-bp PCR product (p4CMProt- p4CMBrot)<br>Primer p4CMProt: CCCATATGGTCGACCTGCAGTTACTTTGATACTATTTCTT<br>Primer p4CMBrot: ACGGGGTGGTGCGTGGATCCTCAATGAACCCTCTTAATTG
p4CM-ptun-3	p4CM + the 1428-bp PCR product (p4CMProt- p4CMBro200t)<br>Primer p4CMProt: CCCATATGGTCGACCTGCAGTTACTTTGATACTATTTCTT<br>Primer p4CMBro200t : ACGGGGTGGTGCGTGGATCCTATGACGTTTTTAAAAAATAACAG
p4CM-ptun-4	p4CM + the 1837-bp PCR product (p4CMP1/2rot- p4CMBrot)<br>Primer p4CMP1/2rot: CCCATATGGTCGACCTGCAGATCTATAGCTGTGTATTTAA<br>Primer p4CMBrot: ACGGGGTGGTGCGTGGATCCTCAATGAACCCTCTTAATTG

Supplementary Figure S3. Determination of the minimal replicon for the chromids. (a) Map of the shuttle vector pOriT-4CM used. The *OriT* gene was the conjugative transfer initiation origin. A region (between the *BamH*I and *Pst*I cleavage sites) containing the replication protein gene *repA* and its flanking sequences was responsible for the replication of the plasmid in *Pseudoalteromonas*. This region was replaced by sequences from chromids in the minimal replicon experiment. *CmR* was the chloromycetin resistant gene and *AmpR* was the ampicillin resistant gene, both of which are used as selective marker genes. The phage f1 region was responsible for the replication of the plasmid in *E. coli*. (b and c) The tested

fragments containing the *ori2* sites and the flanking sequences from the chromids of *P. tunicata* DSM 14096^T (b) and *P. spongiae* JCM 12884^T (c). Genes are shown as blue arrows and *ori2* sites are shown as a red circle. Gene names are shown on the top of the diagram and gene lengths are shown under the diagram with the unit bp. The names of recombinant plasmids containing these fragments are shown on the left and the length of these fragments are shown below. The abilities of the derivatives to replicate in *P. tunicata* and *P. spongiae* are indicated as "+" (yes) or "-" (no). (d) Strains, plasmids and primer sequences used in minimal replicon experiments.
