# Supplementary material for: Evolutionary Trajectory of the Replication Mode of Bacterial Replicons
Source: mBio. 2021 Jan 26;12(1):e02745-20. doi: 10.1128/mBio.02745-20 (PMC7858055; doi:10.1128/mBio.02745-20)
Supplement: FIG S4 [file mBio.02745-20-sf004.pdf]

a

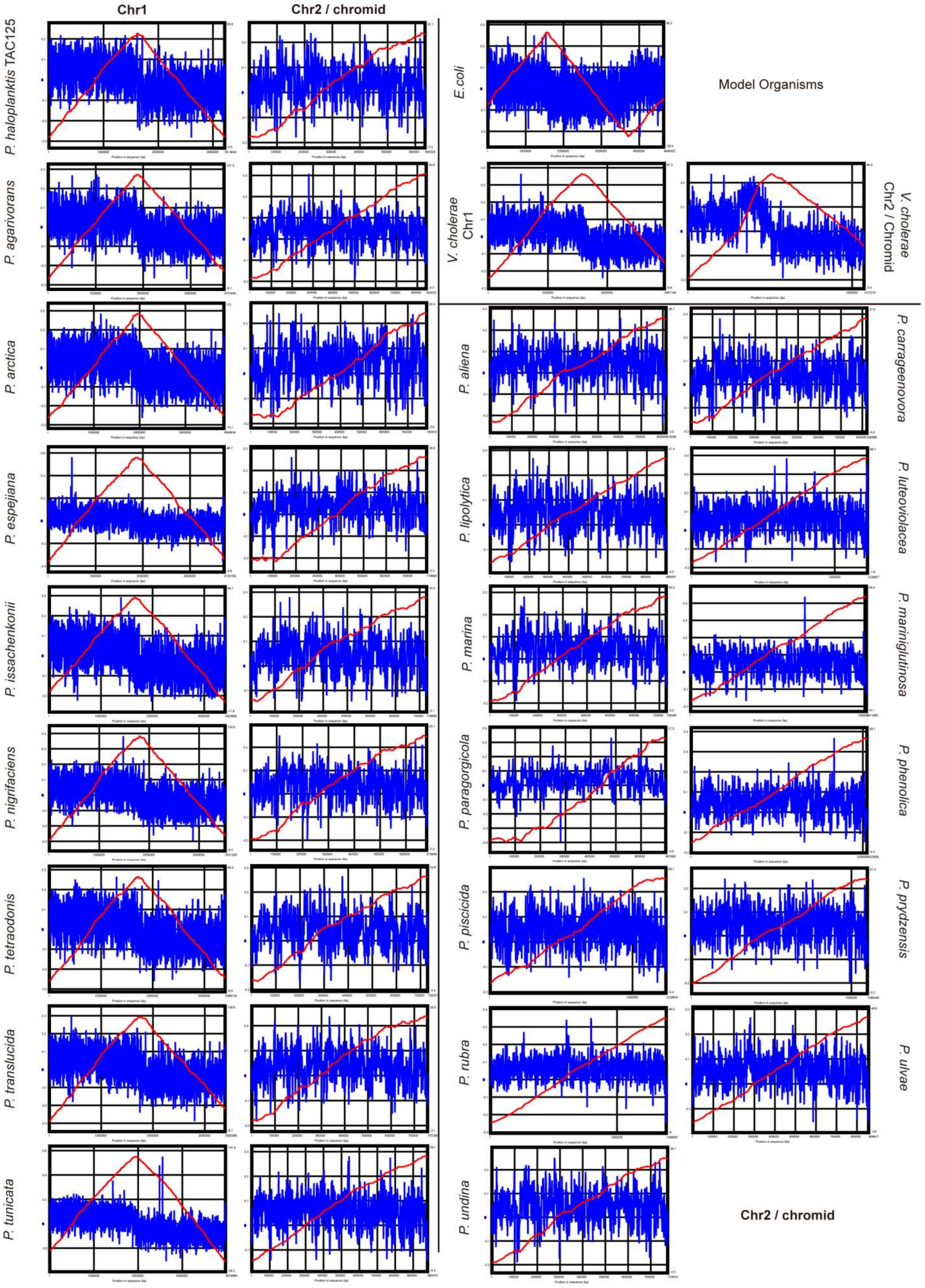
Chr1
Chr2 / chromid
Model Organisms
P. haloplanktis TAC125
P. agarivorans
P. arctica
P. espejiana
P. issachenkonii
P. nigrifaciens
P. tetraodonis
P. translucida
P. tunicata
E.coli
V. cholerae Chr1
V. cholerae Chr2 / Chromid
P. aliena
P. carrageenovora
P. lipolytica
P. luteoviolacea
P. marina
P. mariniglutinosa
P. paragorgicola
P. phenolica
P. piscicida
P. prydzensis
P. rubra
P. ulvae
P. undina
Chr2 / chromid

b

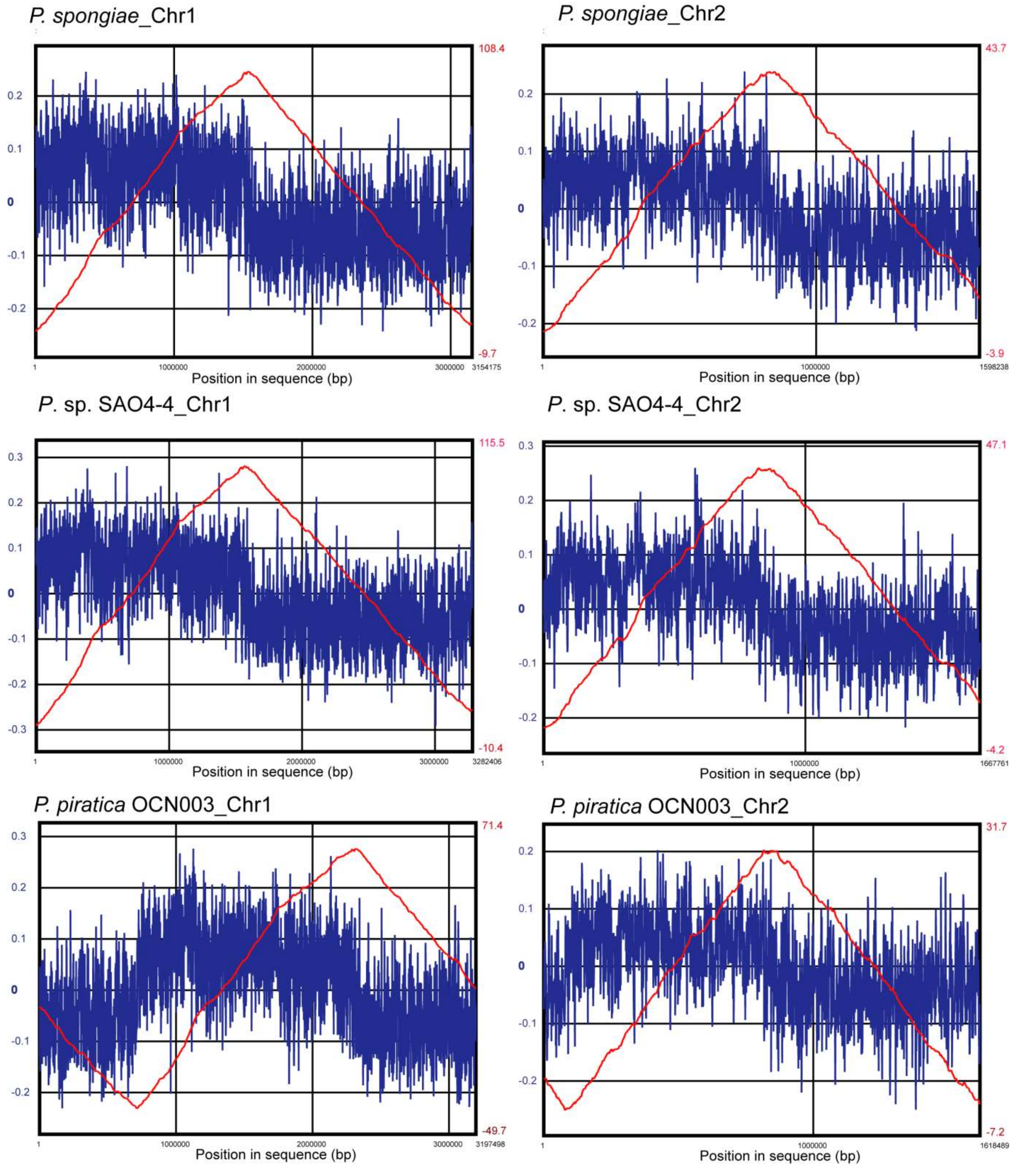

Supplementary Figure S4. (a) Unidirectional replication of *Pseudoalteromonas* chromids revealed by GC skew analyses. (b) Bidirectional replication of *Pseudoalteromonas* chromids revealed by GC skew analyses. For the normal GC skew (blue), the turn point where the skew values turn from negative to positive is approximately at the origin region for replication and the opposite is approximately at the terminus region. For the cumulative GC skew (red), the highest peak is approximately at the terminus region and the lowest peak is approximately at the origin region. Chr1, the main chromosome; Chr2, chromid.
