# Supplementary material for: Evolutionary Trajectory of the Replication Mode of Bacterial Replicons
Source: mBio. 2021 Jan 26;12(1):e02745-20. doi: 10.1128/mBio.02745-20 (PMC7858055; doi:10.1128/mBio.02745-20)
Supplement: FIG S5 [file mBio.02745-20-sf005.pdf]

a

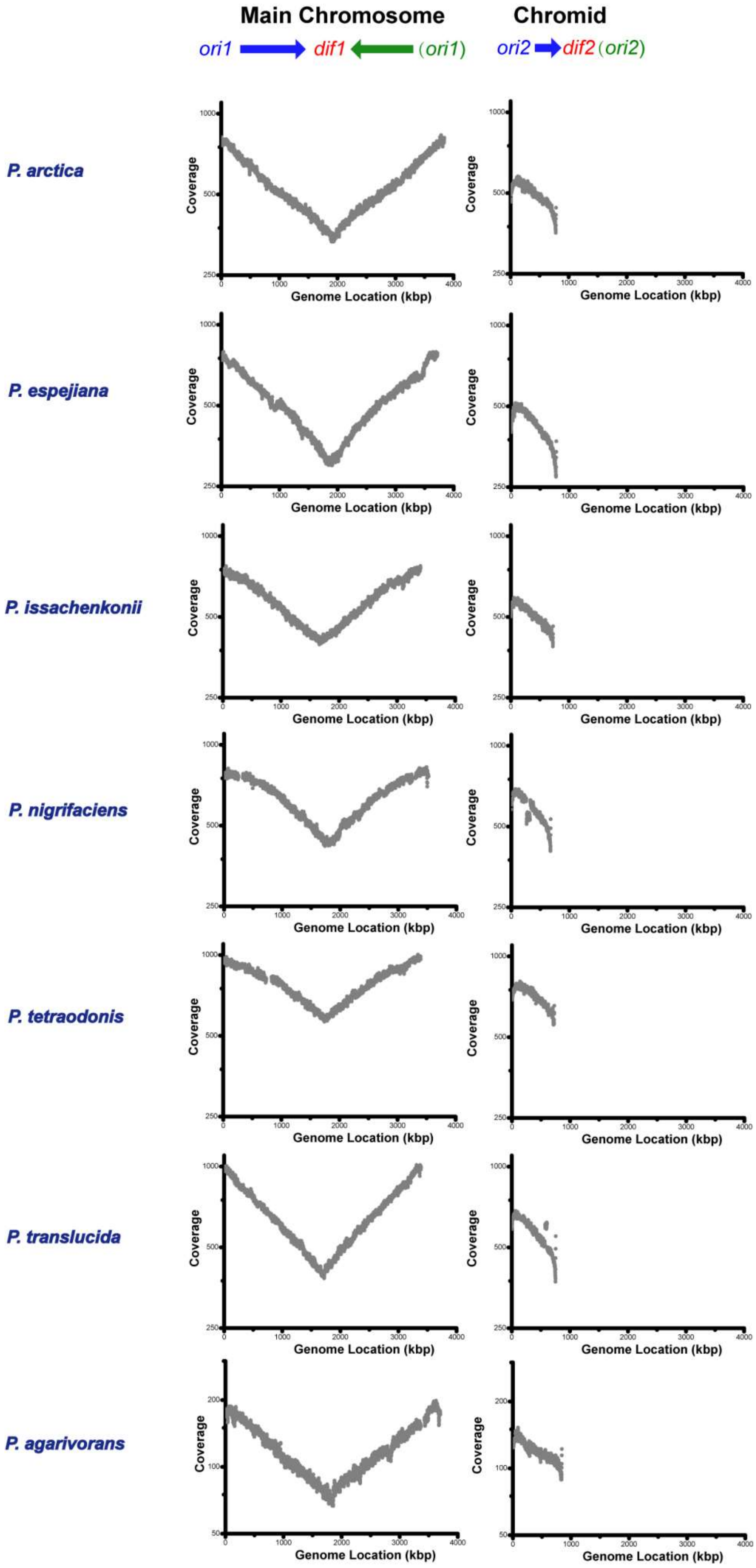

Main Chromosome
ori1 dif1 (ori1)
Chromid
ori2 dif2 (ori2)
P. arctica
P. espejiana
P. issachenkonii
P. nigrifaciens
P. tetraodonis
P. translucida
P. agarivorans
Coverage
Genome Location (kbp)

b

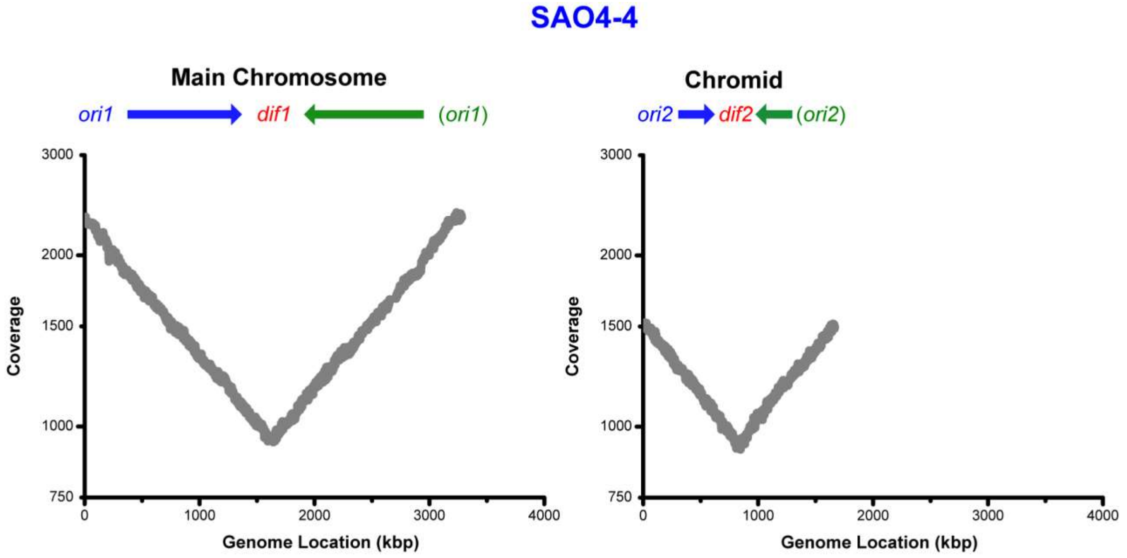

Supplementary Figure S5. (a) Unidirectional replication revealed by deep sequencing at the exponential phase for seven species. (b) Bidirectional replication revealed by deep sequencing at the exponential phase for the strain SAO4-4. Data were presented in bins of 1,000 bp. Note that the y-axis was set to a base-2 logarithmic scale. Arrows indicate the replication direction (blue for clockwise and green for counter clockwise).
