# Supplementary material for: Evolutionary Trajectory of the Replication Mode of Bacterial Replicons
Source: mBio. 2021 Jan 26;12(1):e02745-20. doi: 10.1128/mBio.02745-20 (PMC7858055; doi:10.1128/mBio.02745-20)
Supplement: FIG S6 [file mBio.02745-20-sf006.pdf]

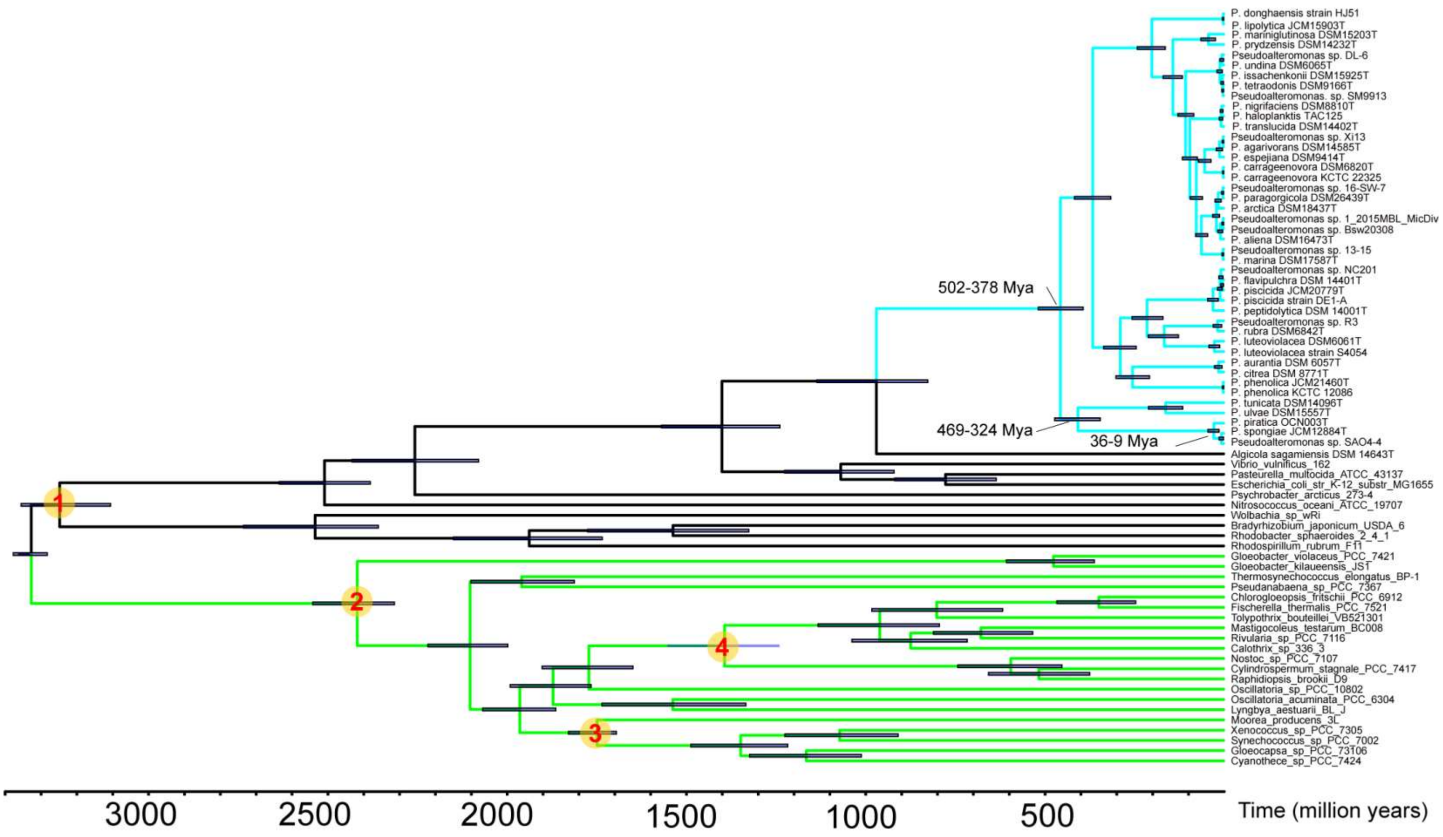

Supplementary Figure S6. Estimated divergence time of *Pseudoalteromonas*. The branches in light blue and green denote *Pseudoalteromonas* and cyanobacteria, respectively. Outgroup species are represented by black branches. Yellow circles on the nodes denote the calibration points, which correspond to the time of the split of α-/γ-proteobacteria (node 1, 2,620-2,360 million years ago, Mya), the origin of cyanobacteria (node 2, 3,000 to 2,320 Mya), the origin of the *Pleurocapsales* (node 3, 1,900-1,700 Mya), and the origin of the *Nostocales* (node 4, 2,450-1,750 Mya), respectively. Horizontal bars on the nodes denote the posterior 95% highest posterior density (HPD) credibility interval (CI).
