# Supplementary material for: Evolutionary Trajectory of the Replication Mode of Bacterial Replicons
Source: mBio. 2021 Jan 26;12(1):e02745-20. doi: 10.1128/mBio.02745-20 (PMC7858055; doi:10.1128/mBio.02745-20)
Supplement: TABLE S4 [file mBio.02745-20-st004.pdf]

Table S4. (a) Integrons, insertion sequences and prophages predicted on *Pseudoalteromonas* chromids. (b) Homologs of *dif2* sites on chromids (Chr2) of *P. spongiae* JCM12884^T, *Pseudoalteromonas* sp. SAO4-4, *P. piratica* OCN003^T and *P. prydzensis* DSM14232^T predicted by BLASTN searches (E-value cutoff of 1).

a

Strains	IS/Integron	Type/Number	Locus_start	Locus_end	Length (bp)
P. spongiae JCM12884^T	Integron	CALIN	1227193	1229278	2086
		CALIN	1461649	1462379	731
		CALIN	1487638	1488514	877
		CALIN	1521501	1522616	1116
	IS	ISNCY	453004	453213	210
		IS200/IS605 ssgr IS200	563141	563497	357
		IS110 ssgr IS1111	622457	622212	246
		Tn3	665252	666139	888
		Tn3	919700	918819	882
		ISNCY	1131487	1131287	201
		Tn3	1288518	1287682	837
		Tn3	1293883	1293251	633
	Prophage	Prophage_1	420991	441132	20142
		Prophage_2	782764	830886	48123
		Prophage_3	1007885	1048498	40614
		Prophage_4	1423301	1538140	114840
Pseudoalteromonas sp. SAO4-4	Integron	CALIN	180150	181211	1062
		CALIN	669480	670481	1002
		CALIN	1261566	1262924	1359
		CALIN	1524405	1525138	734
	IS	ISNCY	459611	459820	210
		IS110 ssgr IS1111	673064	672021	1044
		Tn3	682510	683397	888
		IS110 ssgr IS1111	725945	725163	783
		IS110 ssgr IS1111	726205	725942	264
		IS110 ssgr IS1111	728306	727263	1044
		Tn3	938031	937144	888
		ISNCY	1139484	1139284	201
		IS110 ssgr IS1111	1261446	1260403	1044
		Tn3	1314912	1314076	837
		IS481	1380129	1380479	351
		IS3 ssgr IS3	1381222	1380404	819
	Prophage	Prophage_1	510149	598944	88796
		Prophage_2	805209	848055	42847
		Prophage_3	1015306	1063287	47982
		Prophage_4	1491078	1588732	97655
P. piratica OCN003^T	Integron	CALIN	169318	170204	887
		CALIN	206128	207493	1366
	IS	None IS annotated			
	Prophage	Prophage_1	15857	29549	13693
		Prophage_2	129535	214720	85186
		Prophage_3	623389	658949	35561
		Prophage_4	832663	882535	49873
		Prophage_5	1187334	1204023	16690
		Prophage_6	1523474	1594850	71377
P. agarivorans DSM14585^T	Integron	CALIN	173494	176554	3061
		CALIN	625761	626812	1052
	IS	IS3 ssgr IS407	184446	184712	267

		IS3 ssgr IS407	184793	185560	768
		IS110	281562	280525	1038
		Tn3	326375	327214	840
		IS110	366462	366247	216
		IS110	367009	366485	525
		IS3 ssgr IS3	367608	367847	240
		IS3 ssgr IS3	367956	368786	831
		Tn3	451079	451975	897
		IS110 ssgr IS1111	462438	461284	1155
		IS3 ssgr IS51	466315	465602	714
		IS3 ssgr IS51	466821	466501	321
		IS3 ssgr IS407	475075	473048	2028
		Tn3	499658	500545	888
	Prophage	Prophage_1	173494	191737	18244
		Prophage_2	267254	335025	67772
		Prophage_3	463138	488056	24919
		Prophage_4	627508	666449	38942
		Prophage_5	707934	750100	42167
P. aliena DSM16473^T	Integron	complete	575451	585498	10048
	IS	IS30	18922	17765	1158
		IS3 ssgr IS3	18967	19887	921
		IS3 ssgr IS3	128880	129191	312
		IS3 ssgr IS3	129251	130060	810
		IS1634	134716	136335	1620
		ISL3	233214	234926	1713
		ISL3	234889	235899	1011
		ISL3	237574	240906	3333
		IS701 ssgr ISAba11	299404	298898	507
		IS30	301896	300931	966
		Tn3	324630	324112	519
		IS91	340683	341522	840
		IS91	341524	342648	1125
		IS1182	344514	345581	1068
		IS1182	356760	357302	543
		IS110 ssgr IS1111	359256	358648	609
		Tn3	392615	392025	591
		IS91	397701	398189	489
		IS91	398186	399274	1089
		IS630	417225	416851	375
		IS630	417590	417222	369
		IS21	417629	418336	708
		IS256	430031	429432	600
		IS256	430682	430032	651
		Tn3	455261	456154	894
		Tn3	473195	472317	879
		IS91	576413	575451	963
		IS91	576724	577563	840
		IS91	577565	578689	1125
		IS30	586216	585530	687
		IS30	587381	586416	966
		IS3 ssgr IS3	672643	671834	810
		IS3 ssgr IS3	673014	672703	312
		IS5 ssgr IS427	758210	757710	501
	Prophage	Prophage_1	386643	416140	29498
		Prophage_2	657977	700414	42438

P. arctica DSM18437^T	Integron	CALIN	305783	310081	4299
		complete	532548	538122	5575
	IS	IS110 ssgr IS1111	57446	58459	1014
		IS3 ssgr IS3	113100	112084	1017
		IS3 ssgr IS3	113630	113124	507
		IS30	113994	114962	969
		IS3 ssgr IS3	127298	126468	831
		IS3 ssgr IS3	127648	127337	312
		ISNCY	127696	129417	1722
		IS1182	211864	213198	1335
		Tn3	253707	254546	840
		Tn3	286130	285612	519
		IS1182	307293	308831	1539
		Tn3	351719	351180	540
		IS3 ssgr IS3	359915	359085	831
		IS3 ssgr IS3	360265	359954	312
		Tn3	393213	394106	894
		IS91	405055	405888	834
		IS91	405885	406295	411
		IS91	406408	407085	678
		Tn3	415504	414638	867
		IS91	533477	532548	930
		IS91	533831	534664	834
		IS91	534661	535752	1092
	Prophage	Prophage_1	229207	266256	37050
		Prophage_2	406198	454573	48376
		Prophage_3	533831	572144	38314
P. carrageenovora DSM6820^T	Integron	CALIN	166136	169510	3375
		CALIN	345157	346476	1320
		CALIN	601345	601827	483
	IS	IS110 ssgr IS1111	165991	164837	1155
		Tn3	341093	341932	840
		IS110 ssgr IS1111	345029	343875	1155
		IS110	443754	442498	1257
		Tn3	472159	473055	897
		Tn3	485043	484165	879
		IS91	599240	598515	726
		IS110 ssgr IS1111	603138	602134	1005
	Prophage	Prophage_1	260530	340808	80279
		Prophage_2	683166	725130	41965
P. espejiana DSM9414^T	Integron	CALIN	44676	44740	65
		CALIN	208446	210374	1929
		CALIN	466249	466354	106
		CALIN	580940	581418	479
	IS	IS5 ssgr IS427	35145	35903	759
		IS5 ssgr IS427	167861	168619	759
		Tn3	317725	318564	840
		Tn3	454758	455654	897
		IS5 ssgr IS427	474206	474679	474
		IS5 ssgr IS427	475859	475101	759
		IS256	523998	523321	678
		IS256	524528	524025	504
		IS4 ssgr IS4	525015	524572	444

		IS4 ssgr IS4	525333	525073	261
		IS4 ssgr IS4	525580	525362	219
		IS4 ssgr IS4	525896	525606	291
	Prophage	Prophage_1	38705	71421	32717
		Prophage_2	463126	551949	88824
		Prophage_3	643547	688223	44677
		Prophage_4	717514	738936	21423
P. issachenkonii DSM15925^T	Integron	CALIN	380468	381750	1283
		CALIN	625845	626695	851
	IS	No IS found			
	Prophage	Prophage_1	72510	100615	28106
		Prophage_2	239667	323407	83741
		Prophage_3	596849	646690	49842
P. lipolytica JCM15903^T	Integron	CALIN	707122	708509	1388
		CALIN	797490	798023	534
	IS	Tn3	111349	111906	558
		Tn3	111973	112470	498
		IS4 ssgr IS4	323146	321818	1329
		Tn3	445125	446036	912
		Tn3	747474	746614	861
	Prophage	Prophage_1	586631	616900	30270
		Prophage_2	703138	759553	56416
P. luteoviolacea DSM6061^T	Integron	No Integron found			
	IS	IS91	91153	90320	834
		IS91	91401	91117	285
		IS91	92288	91398	891
		IS3 ssgr IS3	351485	351772	288
		IS3 ssgr IS3	351802	352635	834
		IS30	352659	353363	705
		IS30	366374	366601	228
		IS3 ssgr IS3	373033	372200	834
		IS3 ssgr IS3	373350	373063	288
		IS30	376525	375533	993
		IS3 ssgr IS3	379952	379338	615
		IS3 ssgr IS3	380500	380192	309
		IS5 ssgr IS903	457812	458474	663
		IS3 ssgr IS3	551057	550254	804
		IS3 ssgr IS3	551404	551117	288
		IS5 ssgr IS903	629531	630433	903
		IS3 ssgr IS3	701876	701043	834
		IS3 ssgr IS3	702193	701906	288
		IS30	733832	734212	381
		IS30	734269	734727	459
		IS3 ssgr IS407	826288	825551	738
		IS3 ssgr IS407	826629	826363	267
		ISAs1	862204	861083	1122
		IS3 ssgr IS3	897847	898134	288
		IS3 ssgr IS3	898164	898997	834
		Tn3	920034	920924	891
		IS3 ssgr IS3	1091179	1091466	288
		IS3 ssgr IS3	1091496	1092329	834
		IS5 ssgr IS903	1119053	1118151	903
		IS3 ssgr IS3	1189342	1189629	288

		IS3 ssgr IS3	1189689	1190492	804
		IS3 ssgr IS407	1217973	1217236	738
		IS3 ssgr IS407	1218314	1218048	267
	Prophage	Prophage_1	329705	388292	58588
		Prophage_2	509469	562513	53045
		Prophage_3	678407	719029	40623
P. mariniglutinosa DSM15203^T	Integron	In0	418442	419383	942
	IS	IS3 ssgr IS3	265505	265792	288
		IS3 ssgr IS3	265834	266661	828
		IS21	275235	274480	756
		IS21	276797	275247	1551
		IS91	419383	418442	942
		IS110 ssgr IS1111	421789	420746	1044
		Tn3	633211	634098	888
		Tn3	663343	662486	858
	Prophage	Prophage_1	305064	360485	55422
		Prophage_2	769051	779470	10420
P. marina DSM17587^T	Integron	CALIN	375945	376784	840
		CALIN	518640	520713	2074
		CALIN	597195	600058	2864
		CALIN	602941	603858	918
	IS	Tn3	299475	300314	840
		Tn3	320676	320149	528
		Tn3	406691	407584	894
		IS110	517855	516899	957
		IS110 ssgr IS1111	601385	602539	1155
		IS3 ssgr IS3	705663	704914	750
		IS3 ssgr IS3	706016	705732	285
	Prophage	Prophage_1	220356	283627	63272
		Prophage_2	515885	522921	7037
		Prophage_3	603361	625977	22617
P. nigrifaciens DSM8810^T	Integron	No Integron found			
	IS	IS30	6277	7245	969
		IS66	25670	26011	342
		IS66	26008	26358	351
		IS66	26439	27920	1482
		IS200/IS605	75434	75021	414
		IS200/IS605	75472	76686	1215
		IS200/IS605	88546	90063	1518
		IS110	92433	91450	984
		IS66 ssgr ISBst12	101221	102660	1440
		IS110	115625	116608	984
		IS200/IS605 ssgr IS200	148766	149197	432
		ISAs1	156157	155021	1137
		IS66	175480	173936	1545
		IS66	175825	175520	306
		IS66	176175	175870	306
		IS200/IS605 ssgr IS1341	243352	242126	1227
		IS200/IS605	243391	243786	396
		IS21	260988	260233	756
		IS21	262526	261003	1524
		IS200/IS605	309241	308855	387
		IS66	309397	309702	306

		IS66	309747	310052	306
		IS66	310092	311636	1545
		IS200/IS605 ssgr IS1341	311711	312952	1242
		IS21	324298	323543	756
		IS21	325836	324313	1524
		Tn3	360179	361069	891
		Tn3	375980	375117	864
		IS200/IS605 ssgr IS1341	381717	382943	1227
		IS3 ssgr IS3	457327	456518	810
		IS3 ssgr IS3	457698	457387	312
		IS110	488230	488006	225
		IS110 ssgr IS1111	488951	488211	741
		IS200/IS605	575731	575240	492
		IS200/IS605 ssgr IS1341	575770	576996	1227
		IS30	604562	603594	969
	Prophage	Prophage_1	221025	271984	50960
		Prophage_2	477191	516316	39126
		Prophage_3	547224	565246	18023
		Prophage_4	626624	644305	17682
P. paragorgicola DSM26439^T	Integron	complete	410224	413261	3038
	IS	IS481	52515	51475	1041
		IS110	98609	97626	984
		IS481	129774	130202	429
		IS481	130587	131108	522
		IS4 ssgr IS4	149212	150540	1329
		IS481	161422	161850	429
		IS481	162235	162756	522
		IS110	196382	195345	1038
		IS481	196500	197540	1041
		Tn3	279460	280353	894
		Tn3	295213	294347	867
		IS91	411192	410224	969
		IS110 ssgr IS1111	414029	413568	462
		IS110 ssgr IS1111	414723	414103	621
		IS4 ssgr IS4	441653	442981	1329
		IS481	498373	497852	522
		IS110	523637	524674	1038
		IS3 ssgr IS3	526699	526968	270
		IS481	558424	557903	522
	Prophage	Prophage_1	437429	466499	29071
		Prophage_2	517398	559551	42154
P. phenolica JCM21460^T	Integron	In0	554180	555112	933
		CALIN	563344	572686	9343
		CALIN	682191	683360	1170
	IS	No IS found			
	Prophage	Prophage_1	539966	603924	63959
		Prophage_2	663920	736843	72924
P. piscicida JCM20779^T	Integron	No Integron found			
	IS	ISNCY	21418	21630	213
		IS3 ssgr IS3	256778	255948	831
		IS3 ssgr IS3	257164	256817	348
		IS1182	535866	537212	1347
		IS110 ssgr IS1111	554312	553302	1011

		IS3 ssgr IS3	645818	646648	831
		Tn3	721464	722420	957
		Tn3	736804	737685	882
		IS1182	813273	814619	1347
		IS91	895394	894819	576
		IS1182	1063194	1064540	1347
		IS3 ssgr IS3	1079283	1078453	831
		IS3 ssgr IS3	1079633	1079322	312
		IS3 ssgr IS3	1081985	1082332	348
		IS3 ssgr IS3	1082371	1083201	831
		IS3 ssgr IS407	1175512	1174775	738
		IS3 ssgr IS407	1175853	1175587	267
		IS3 ssgr IS3	1228859	1229170	312
		IS3 ssgr IS3	1229209	1230039	831
	Prophage	Prophage_1	98022	136803	38782
		Prophage_2	205346	311067	105722
		Prophage_3	507582	542744	35163
		Prophage_4	849202	887238	38037
		Prophage_5	1078453	1090513	12061
P. prydzensis DSM14232^T	Integron	CALIN	415204	417319	2116
	IS	Tn3	677553	678440	888
		New_Family	700434	700306	129
		Tn3	712569	711712	858
		IS110	987127	988164	1038
	Prophage	Prophage_1	137535	177390	39856
		Prophage_2	305138	349002	43865
		Prophage_3	743277	760930	17654
		Prophage_4	831629	842891	11263
		Prophage_5	987127	1026439	39313
P. rubra DSM6842^T	Integron	No Integron found			
	IS	IS110 ssgr IS1111	52063	53076	1014
		Tn3	1124601	1125491	891
		Tn3	1370323	1371165	843
	Prophage	Prophage_1	235264	280261	44998
		Prophage_2	997322	1072648	75327
P. tetraodonis DSM9166^T	Integron	CALIN	18739	20621	1883
		CALIN	371140	373181	2042
		CALIN	517799	518964	1166
		CALIN	625721	626939	1219
	IS	ISL3	210932	212545	1614
		ISL3	214158	217478	3321
		IS256	296929	298131	1203
		Tn3	308371	307853	519
		IS256	352715	353917	1203
		Tn3	359491	358901	591
		Tn3	398207	399100	894
		IS3 ssgr IS51	413432	412536	897
		IS3 ssgr IS51	413755	413435	321
		Tn3	422553	421699	855
	Prophage	Prophage_1	252020	309118	57099
P. translucida DSM14402^T	Integron	CALIN	343393	345166	1774
	IS	IS200/IS605	76527	78044	1518

		IS66	176134	174572	1563
		IS66	176578	176231	348
		IS66	176874	176575	300
		ISL3	188908	190398	1491
		IS3 ssgr IS3	204701	203892	810
		IS3 ssgr IS3	205072	204761	312
		IS3 ssgr IS407	345310	345576	267
		IS3 ssgr IS407	345570	346424	855
		IS110	346900	346436	465
		IS110	348212	346953	1260
		Tn3	406691	407581	891
		Tn3	422497	421634	864
		IS4 ssgr IS4	510437	509112	1326
		IS4 ssgr IS4	573269	571938	1332
		IS4 ssgr IS4	607625	606294	1332
		IS200/IS605	679989	679306	684
		IS200/IS605	680531	680016	516
	Prophage	Prophage_1	36905	51927	15023
		Prophage_2	241213	275558	34346
		Prophage_3	542820	584314	41495
		Prophage_4	602479	654003	51525
P. tunicata DSM14096^T	Integron	CALIN	58742	59768	1027
		CALIN	105318	106594	1277
		CALIN	188086	188786	701
	IS	IS1182	107055	106804	252
		IS1182	188987	189367	381
		IS1182	189759	190397	639
		IS110	350604	349846	759
		IS110 ssgr IS1111	354798	353779	1020
		IS3 ssgr IS3	359696	358872	825
		IS4 ssgr IS10	360120	361313	1194
		IS110 ssgr IS1111	601353	602372	1020
		Tn3	668949	669860	912
		IS91	912372	911905	468
		IS91	912866	912369	498
		IS110	915629	914985	645
		IS1182	917010	917276	267
		IS1182	917284	918447	1164
		IS1182	920743	921603	861
		IS1182	921561	922295	735
		IS110	928502	927828	675
	Prophage	Prophage_1	170473	221918	51446
		Prophage_2	353119	415525	62407
		Prophage_3	732133	787473	55341
		Prophage_4	905763	948671	42909
P. ulvae DSM15557^T	Integron	CALIN	179251	179388	138
		CALIN	190328	191903	1576
		CALIN	387500	389031	1532
		CALIN	517573	518954	1382
	IS	IS3 ssgr IS51	100306	100584	279
		IS3 ssgr IS51	100623	101453	831
		IS91	189498	189076	423
		IS110	192890	191940	951
		IS110	379634	380059	426

		IS3 ssgr IS51	463503	462673	831
		IS3 ssgr IS51	463820	463542	279
		IS110	517244	516294	951
		Tn3	589148	590035	888
		IS3 ssgr IS51	640717	640995	279
		IS3 ssgr IS51	641034	641864	831
	Prophage	Prophage_1	340398	395066	54669
		Prophage_2	646168	677362	31195
P. undina DSM6065^T	Integron	CALIN	645686	648106	2421
		CALIN	679415	680736	1322
	IS	No IS found			
	Prophage	Prophage_1	240362	296883	56522
		Prophage_2	450014	502771	52758
		Prophage_3	675032	683984	8953

b

Strains	Start	End	String	Note	BLASTN searches with *dif2* sites of different strains as the reference								
					Identities	Length_ matched	Mismatch	Gap	Start	End	E_value	Score	Ref. strains
P. spongiae JCM12884^T	453266	453278	+	adjacent to Prophage_1 (420991-441132), distance ~12 kb	100.00	13	0	0	7	19	0.32	26.3	*P. haloplanktis* TAC125
	750982	750994	-		100.00	13	0	0	4	16	0.32	26.3	*P. haloplanktis* TAC125
	819264	819291	-	***dif2* site, inside Prophage 2 (782764-830886)**	100.00	28	0	0	1	28	4.00E-10	56	*P. spongiae* JCM12884^T
	814906	814918	-	inside Prophage_2 (782764-830886)	100.00	13	0	0	13	25	0.32	26.3	*P. phenolica* JCM21460^T
	984606	984618	-		100.00	13	0	0	8	20	0.32	26.3	*Pseudoalteromonas* sp. SAO4-4
	994493	994509	-	adjacent to Prophage_3 (1007885-1048498), distance ~13 kb	94.12	17	1	0	4	20	0.32	26.3	*P. tunicata* DSM14096^T
	1018030	1018042	-	inside Prophage_3 (1007885-1048498)	100.00	13	0	0	7	19	0.32	26.3	*P. haloplanktis* TAC125
	1047160	1047172	-	inside Prophage_3 (1007885-1048498)	100.00	13	0	0	3	15	0.32	26.3	*P. tunicata* DSM14096^T
	1464816	1464828	-	inside Prophage_4 (1423301-1538140)	100.00	13	0	0	1	13	0.32	26.3	*P. piratica* OCN003^T
	51368	51381	-		100.00	14	0	0	15	28	0.08	28.2	*P. spongiae* JCM12884^T
	127614	127627	-		100.00	14	0	0	2	15	0.08	28.2	*P. marina* DSM17587^T
	199851	199867	+		94.12	17	1	0	9	25	0.32	26.3	*Pseudoalteromonas* sp. SAO4-4
	299329	299342	+		100.00	14	0	0	1	14	0.08	28.2	*P. undina* DSM6065^T
	345036	345048	+		100.00	13	0	0	12	24	0.32	26.3	*Pseudoalteromonas* sp. SAO4-4
	492018	492030	-		100.00	13	0	0	1	13	0.32	26.3	*P. haloplanktis* TAC125
	511629	511641	-		100.00	13	0	0	8	20	0.32	26.3	*P. haloplanktis* TAC125
	562331	562343	-		100.00	13	0	0	12	24	0.32	26.3	*P. phenolica* JCM21460^T
	909080	909093	+		100.00	14	0	0	2	15	0.08	28.2	*P. lipolytica* JCM15903^T
	1138509	1138521	+		100.00	13	0	0	8	20	0.32	26.3	*P. nigrifaciens* DSM8810^T
Pseudoalteromonas sp. SAO4-4	517053	517065	-	inside Prophage_1 (510149-598944)	100.00	13	0	0	8	20	0.33	26.3	*P. haloplanktis* TAC125
	754988	755005	-		94.44	18	1	0	7	24	0.084	28.2	*P. piscicida* JCM20779^T
	772606	772618	-		100.00	13	0	0	4	16	0.33	26.3	*P. haloplanktis* TAC125
	828218	828245	-	***dif2* site, inside Prophage 2 (805209-848055)**	100.00	28	0	0	1	28	4.00E-10	56	*Pseudoalteromonas* sp. SAO4-4
	986092	986104	-		100.00	13	0	0	8	20	0.33	26.3	*Pseudoalteromonas* sp. SAO4-4
	997300	997316	-	adjacent to Prophage_3 (1015306-1063287), distance ~18 kb	94.12	17	1	0	4	20	0.33	26.3	*P. tunicata* DSM14096^T
	1028608	1028620	-	inside Prophage_3 (1015306-1063287)	100.00	13	0	0	7	19	0.33	26.3	*P. haloplanktis* TAC125
	1549673	1549685	+	inside Prophage_4 (1491078-1588732)	100.00	13	0	0	6	18	0.33	26.3	*P. haloplanktis* TAC125
	53134	53147	-		100.00	14	0	0	15	28	0.084	28.2	*Pseudoalteromonas* sp. SAO4-4
	123701	123714	-		100.00	14	0	0	2	15	0.084	28.2	*P. marina* DSM17587^T
	195154	195170	+		94.12	17	1	0	9	25	0.33	26.3	*Pseudoalteromonas* sp. SAO4-4
	305372	305385	+		100.00	14	0	0	1	14	0.084	28.2	*P. arctica* DSM18437^T
	351082	351094	+		100.00	13	0	0	12	24	0.33	26.3	*Pseudoalteromonas* sp. SAO4-4
	459873	459885	+		100.00	13	0	0	7	19	0.33	26.3	*P. haloplanktis* TAC125
	925808	925821	+		100.00	14	0	0	2	15	0.084	28.2	*P. lipolytica* JCM15903^T
	1316539	1316551	-		100.00	13	0	0	5	17	0.33	26.3	*P. marina* DSM17587^T
P. piratica OCN003^T	164235	164251	+	inside Prophage_2 (129535-214720)	94.12	17	1	0	5	21	0.32	26.3	*P. tunicata* DSM14096^T
	607740	607752	-	adjacent to Prophage_3 (623389-658949), distance ~16 kb	100.00	13	0	0	1	13	0.32	26.3	*P. piratica* OCN003^T
	632938	632950	+	inside prophage_3 (623389-658949)	100.00	13	0	0	3	15	0.32	26.3	*P. tunicata* DSM14096^T
	851579	851591	+	inside Prophage_4 (832663-882535)	100.00	13	0	0	5	17	0.32	26.3	*P. piratica* OCN003^T
	862562	862589	+	***dif2* site, inside Prophage_4 (832663-882535)**	100.00	28	0	0	1	28	4.00E-10	56	*P. piratica* OCN003^T
	1119575	1119588	-		100.00	14	0	0	1	14	0.081	28.2	*P. tunicata* DSM14096^T
	1149425	1149437	+		100.00	13	0	0	1	13	0.32	26.3	*P. haloplanktis* TAC125
	573778	573791	+		100.00	14	0	0	6	19	0.081	28.2	*P. tunicata* DSM14096^T
	885683	885695	+		100.00	13	0	0	7	19	0.32	26.3	*P. phenolica* JCM21460^T
	983457	983469	+		100.00	13	0	0	6	18	0.32	26.3	*P. agarivorans* DSM14585^T
	999710	999722	+		100.00	13	0	0	1	13	0.32	26.3	*P. ulvae* DSM15557^T
	1057048	1057060	+		100.00	13	0	0	12	24	0.32	26.3	*P. phenolica* JCM21460^T
	1393995	1394008	+		100.00	14	0	0	1	14	0.081	28.2	*P. tetraodonis* DSM9166^T
P. prydzensis DSM14232^T	157012	157023	-	inside Prophage_1 (137535-177390)	100.00	12	0	0	3	14	0.86	24.3	*P. paragorgicola* DSM26439^T
	327858	327873	+	inside Prophage_2 (305138-349002)	93.75	16	1	0	12	27	0.86	24.3	*Pseudoalteromonas* sp. SAO4-4
	764251	764262	-	adjacent to Prophage_3 (743277-760930), distance ~3 kb	100.00	12	0	0	1	12	0.86	24.3	*P. tunicata* DSM14096^T
	841694	841705	-	inside Prophage_4 (831629-842891)	100.00	12	0	0	8	19	0.86	24.3	*Pseudoalteromonas* sp. SAO4-4
	996660	996671	-	inside Prophage_5 (987127-1026439)	100.00	12	0	0	1	12	0.86	24.3	*P. espejiana* DSM9414^T

	1015917	1015928	+	inside Prophage_5 (987127-1026439)	100.00	12	0	0	11	22	0.86	24.3	*P. piscicida* JCM20779^T
	1073622	1073633	-		100.00	12	0	0	16	27	0.86	24.3	*P. prydzensis* DSM14232^T
	1083960	1083987	+	***dif2* site**	100.00	28	0	0	1	28	2.00E-10	56	*P. prydzensis* DSM14232^T
	1085412	1085423	-		100.00	12	0	0	8	19	0.86	24.3	*P. prydzensis* DSM14232^T
	1097555	1097566	+		100.00	12	0	0	16	27	0.86	24.3	*P. tunicata* DSM14096^T
	19797	19809	-		100.00	13	0	0	11	23	0.22	26.3	*P. nigrifaciens* DSM8810^T
	43632	43643	+		100.00	12	0	0	1	12	0.86	24.3	*P. haloplanktis* TAC125
	74844	74855	-		100.00	12	0	0	3	14	0.86	24.3	*P. undina* DSM6065^T
	78187	78198	+		100.00	12	0	0	17	28	0.86	24.3	*P. tunicata* DSM14096^T
	235112	235123	+		100.00	12	0	0	8	19	0.86	24.3	*P. phenolica* JCM21460^T
	244277	244288	-		100.00	12	0	0	8	19	0.86	24.3	*P. phenolica* JCM21460^T
	406065	406076	-		100.00	12	0	0	8	19	0.86	24.3	*P. nigrifaciens* DSM8810^T
	429778	429790	-		100.00	13	0	0	12	24	0.22	26.3	*P. phenolica* JCM21460^T
	449895	449906	-		100.00	12	0	0	9	20	0.86	24.3	*P. tunicata* DSM14096^T
	466901	466912	+		100.00	12	0	0	3	14	0.86	24.3	*P. haloplanktis* TAC125
	493906	493917	+		100.00	12	0	0	4	15	0.86	24.3	*P. tunicata* DSM14096^T
	507984	507995	-		100.00	12	0	0	1	12	0.86	24.3	*P. tetraodonis* DSM9166^T
	534593	534604	-		100.00	12	0	0	17	28	0.86	24.3	*P. prydzensis* DSM14232^T
	551401	551412	-		100.00	12	0	0	9	20	0.86	24.3	*P. piscicida* JCM20779^T
	567966	567978	+		100.00	13	0	0	2	14	0.22	26.3	*P. haloplanktis* TAC125
	580781	580792	-		100.00	12	0	0	10	21	0.86	24.3	*P. nigrifaciens* DSM8810^T
	610782	610793	+		100.00	12	0	0	6	17	0.86	24.3	*P. phenolica* JCM21460^T
	634520	634531	-		100.00	12	0	0	2	13	0.86	24.3	*P. tetraodonis* DSM9166^T
	640876	640887	+		100.00	12	0	0	8	19	0.86	24.3	*P. phenolica* JCM21460^T
	646589	646600	+		100.00	12	0	0	1	12	0.86	24.3	*P. aliena* DSM16473^T
	931249	931261	-		100.00	13	0	0	3	15	0.22	26.3	*P. marina* DSM17587^T
	970782	970794	+		100.00	13	0	0	9	21	0.22	26.3	*P. tunicata* DSM14096^T
	973414	973425	+		100.00	12	0	0	12	23	0.86	24.3	*Pseudoalteromonas* sp. SAO4-4
	985270	985282	-		100.00	13	0	0	7	19	0.22	26.3	*P. nigrifaciens* DSM8810^T
